# Effect of an Online Continuing Professional Development Course on Physicians’ Intention to Approach a Colleague in Difficulty: Mixed Methods Convergent Study

**DOI:** 10.2196/80199

**Published:** 2026-02-05

**Authors:** Florence Lizotte, Martin Tremblay, Caroline Biron, Éloi Lachance, Souleymane Gadio, Roberta de Carvalho Corôa, Claude Bernard Uwizeye, Sam J Daniel, France Légaré

**Affiliations:** 1 Faculty of Medicine Université Laval Québec, QC Canada; 2 Fédération des médecins spécialistes du Québec Montréal, QC Canada; 3 VITAM – Centre de recherche en santé durable Université Laval Québec, QC Canada; 4 Department of Management Université Laval Québec, QC Canada; 5 Faculty of Medicine McGill University Montreal, QC Canada; 6 Centre de recherche du CHU de Québec Université Laval Québec, QC Canada

**Keywords:** burnout, continuing medical education, continuing professional development, distance education, helping behavior, occupational stress, peer support, physicians, psychological well-being, social support

## Abstract

**Background:**

Burnout and psychological distress are prevalent among physicians. Peer support appears to play a protective role, yet little is known about training interventions that motivate physicians to approach peers in difficulty, as such effects are often overlooked or assessed using nonvalidated tools.

**Objective:**

We evaluated the effects of an online continuing professional development (CPD) course designed to increase physicians’ intention to approach a colleague in difficulty.

**Methods:**

Physicians who completed a 1-hour asynchronous online CPD course between March 2022 and May 2024 were invited to participate in this mixed methods convergent study. The e-learning course aimed to increase physicians’ confidence in approaching colleagues in difficulty by recognizing signs of psychological distress, offering support, and referring them to appropriate resources. Participant characteristics were collected, and behavioral intention to approach a colleague in difficulty along with its determinants were measured pre- and postcourse using the validated CPD-REACTION tool. Differences in mean pre-post intention scores were assessed using 2-tailed paired *t* tests (n=466) and generalized estimating equations. Factors associated with postcourse intention were examined using multivariate analysis (n=466). Four months later, the proportion of physicians reporting adoption of the behavior was calculated (n=61). Qualitative responses to open-ended questions were analyzed thematically using behavior change models, and behavior change techniques used in the course were identified. Quantitative and qualitative results were triangulated. We reported results following STROBE (Strengthening the Reporting of Observational Studies in Epidemiology) and SRQR (Standards for Reporting Qualitative Research) guidelines for quantitative and qualitative analyses, respectively.

**Results:**

Among 792 participating physicians, 466 (58.8%) completed online questionnaires pre- and postcourse. The average participant age was 48 (SD 12.4) years; 43.5% (332/762) were women, and 86% (655/762) were specialists. The average precourse intention score was 3.88 (SD 1.73) and average postcourse intention score was 4.92 (SD 1.40), for an adjusted mean difference of 1.06 (95% CI 0.93-1.20; *P*<.001). Factors associated with postcourse intention were beliefs about capabilities (β=0.52; *P*<.001), social influences (β=0.27; *P*<.001), and moral norm (β=0.26; *P*=.03; *R*^2^=0.22). Four months later, 41% (25/61; 95% CI 28.6%-54.3%) of participants reported having approached a colleague in difficulty. Frequently reported reasons for intention to adopt behavior were beliefs about capabilities, beliefs about consequences, and knowledge. Quantitative and qualitative results converged on beliefs about capabilities but diverged regarding beliefs about consequences. A total of 7 behavioral change techniques were identified in the CPD course: goal setting, increasing competence, planning, persuasive communication, behavior-related information, modeling, and behavioral experiments.

**Conclusions:**

This online CPD course increased physicians’ intention to approach a colleague in difficulty. The results highlight beliefs about capabilities as a key determinant of this behavioral intention. The study suggests that online learning has strong potential to raise awareness about peer support and ultimately build a culture of care among health care workers.

## Introduction

Burnout is prevalent among physicians [1-4]. In 2021, the Canadian Medical Association reported that half of Canadian physicians experienced at least 1 symptom of burnout on a weekly basis or more frequently [1]. Physicians experience greater social isolation compared to workers in other sectors [5]. Social isolation is linked to higher levels of burnout in physicians, but the direction of this relationship—whether it is a cause, a consequence, or bidirectional—remains unclear [5]. Furthermore, the “second victim” phenomenon, or health care providers’ distress after a patient adverse event, can lead to burnout [6,7]. A patient adverse event is an unexpected but often preventable event which results in consequences for the patient of varying seriousness, including prolonging treatment, causing discomfort, disability, or death [7]. Moral distress was also associated with burnout in health care workers during the COVID-19 pandemic [8]. Moral distress is experienced when a health care worker “feels unable to act in accordance with core values and obligations, or attempted actions fail to achieve the desired outcome” [9]. Burnout impacts physicians’ health and can have consequences on patient safety, satisfaction, and physician retention [10-12]. Improving physician well-being is important and the well-being of health care and social service workers is one element of the Quintuple Aim framework, whose goals are to improve population health, enhance care experiences, reduce costs, improve clinician well-being, and improve health equity [13].

A 2022 scoping review showed that peer support, informally or formally provided by health care providers with similar work conditions and experiences, can help health care providers after a distressing patient adverse event [14]. Furthermore, physicians themselves appear to find it the best form of support [15,16]. A 2024 trial found a peer support program protected health care providers aged 30 years or younger from psychological distress, although the effect was not significant for all age categories combined [17]. In another study, a peer support program reduced psychological distress among anesthesia professionals [18].

Components of a peer support conversation include initial outreach, listening, reflecting, reframing, eliciting the peer’s personal coping mechanisms, closing the discussion, and referring the peer to available resources [19]. Experts recommend that peer supporters should receive training on listening skills, psychological first aid, and information about referral options to fulfill their role adequately [20]. Many formal peer-support programs have been implemented in health care organizations and include dedicated training courses for peer supporters [15,17,21-49]. Some of these training programs are inspired by (1) the Stress First Aid model, initially developed for high-risk occupations such as the military service [45,47-49]; (2) the psychological first aid training developed by George Everly [22,44]; (3) the peer-support approach developed by Shapiro and Galowitz [27,32,35,37,39] and (4) the Scott Three-Tier Intervention Model of peer support [15,21,23,28,30,41,42]. However, evaluation of the effect of courses about peer support is often omitted in program evaluations or assessed using cross-sectional surveys with nonvalidated measures [15,22,26,27,30,34,38-40,43]. Furthermore, most courses studied are in-person workshops rather than online continuing professional development (CPD) courses [15,22,26,27,30,34,38-40,43]. Therefore, we sought to assess the effect of an online CPD course on the intention of physicians to approach a colleague in difficulty using a mixed methods convergent research design and validated measures. We also analyzed why participants considered approaching a colleague in difficulty by conducting thematic analyses of qualitative data and triangulating these findings with quantitative results.

## Methods

### Study Design

**Figure 1:**
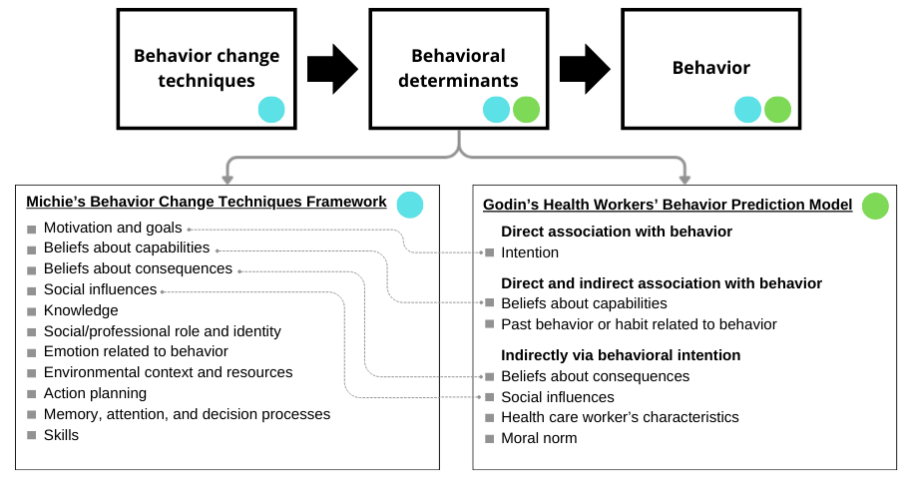
Framework combining Godin’s integrated model for predicting health professionals’ behavior and Michie’s framework on behavior change techniques [52,53].

We used a mixed methods convergent research design to triangulate results obtained from quantitative and qualitative methods [50]. Quantitative analysis involved a quasi-experimental before-and-after study design to evaluate the effect of an online CPD course that is part of the CPD program of the Fédération des médecins spécialistes du Québec (FMSQ)*.* In this design, measurements before the CPD course served as comparators for the same measurements taken after the CPD course for the same participants [50]. We analyzed responses to open questions with a qualitative thematic deductive approach to understand why participants considered integrating the targeted behavior into their professional practice [50]. For both quantitative and qualitative analyses, we used Godin’s integrated model for predicting health professionals’ behavior as a conceptual framework [51] and we used Michie’s conceptual framework on behavior change techniques (BCTs) to explore our qualitative data in detail [52]. As shown in Figure 1 [52,53], these frameworks share the concept that behavioral determinants lead to behavior adoption [51-53]. Both originate from Ajzen’s Theory of Planned Behavior and have many behavioral determinants in common [51-53]. Michie et al [52] propose that BCTs can influence and target behavioral determinants, which in turn translate into behavior change. Possible behavioral change techniques include planning, persuasive communication and others [52]. Details on these 2 conceptual frameworks are available in Multimedia Appendix 1 [51-54]. We reported results following the STROBE (Strengthening the Reporting of Observational Studies in Epidemiology) guideline for quantitative results (Multimedia Appendix 2) [55] and used the SRQR (Standards for Reporting Qualitative Research) for qualitative results (Multimedia Appendix 3) [56].

### Participants and Recruitment

The study took place in Quebec, Canada, from March 1, 2022, to June 20, 2024. Targeted participants were mainly specialists from the FMSQ membership. However, general practitioners who subscribe to the FMSQ’s online learning platform MÉDUSE could also participate in the study. The study population was physicians who registered for the course and completed an online consent form. After registering, participants were informed about the study by a system-generated message displayed at the beginning of the online course. The CPD course was not mandatory but was recognized by the Royal College of Physicians and Surgeons of Canada as a 1-hour group learning credit (Section 1) [57]. This convenience sampling was based on time constraints and access to MÉDUSE. Inclusion criteria were being a licensed physician in Quebec and participating in the online CPD course. There were no exclusion criteria.

### Intervention

We describe the intervention according to the Template for Intervention Description and Replication (TIDieR) guidelines [58]. The intervention was a 1-hour asynchronous online CPD course developed by the FMSQ to help physicians approach colleagues in difficulty (eg, burnout, psychological distress, second victim symptoms). Thus, this course focused on a form of peer support. The term “colleague” included anyone in the health and social services sector, including administrative staff. The scientific committee comprised 3 physicians, 2 of whom had peer support experience through their work with the Québec Physicians’ Health Program (QPHP). The QPHP organization also helped with course design. A scenario-based video, filmed with professional actors, illustrates the QPHP’s 5-Step Caring Approach: (1) becoming aware of context and stressors, (2) recognizing the warning signs, (3) initiating a conversation with the colleague, (4) providing compassionate support, and (5) respecting your own limits and practicing self-care [59]. Approaching a colleague in difficulty corresponds to Tier 1 of the Scott Three-Tier Model of Support, which includes immediate team members of a health care worker experiencing distress [21].

A CPD expert with more than 10 years of experience (MT) used Godin’s integrated model for predicting health professionals’ behavior [51] for instructional design to increase participants’ willingness to approach a colleague in difficulty. For instance, the model was used to increase participants’ beliefs in the consequences of approaching a colleague in difficulty. In the first module of the course, viewers are shown how a compassionate approach, which enables a colleague to share emotions related to stressful situations, can help mitigate the impact of these sources of stress. In the second module, physicians are shown the negative consequences that may occur if a colleague in difficulty is not helped or if help is delayed. Participants completed an intervention plan during the CPD course to use when approaching a colleague at their workplace. Other educational strategies include narrated vignettes, knowledge-check questions, additional readings, and reflective writing, which often refers back to the scenario shown in the video. During the online course, physicians are also provided with a self-evaluation tool to identify when they or a colleague may need help. The tool provides individual strategies to maintain or improve mental health at each step along the continuum from being healthy to having important mental health problems [60].

During the course, participants have the option of downloading 10 summaries (ranging from 1 to 3 pages) of course content or supplementary materials [59-67]. Bloom’s taxonomy defined the course’s learning objectives [68]. The CanMEDS role targeted by this course was the professional role, specifically with the objective of emphasizing the commitment to physicians’ health and well-being, which ultimately promotes optimal patient care [69]. The CanMEDS framework highlights skills that are essential throughout medical practice, providing guidance for medical education and ongoing professional development for practicing physicians, all within a competency-based approach [69]. The course was made available on MÉDUSE, the FMSQ’s online CPD platform [70]. Physicians completing the course were sent an automated reminder after 2 weeks of inactivity, and the unfinished course remained available on the physicians’ dashboard on MÉDUSE as a reminder to complete it.

### Outcomes

The primary outcome was the intention of physicians to approach a colleague in difficulty. Godin defined intention, from the work of Ajzen and Fishbein on the Theory of Reasoned Action, as the motivation to adopt a specific behavior [51]. In this study, this targeted behavior was approaching a colleague in difficulty.

Intention to adopt a behavior is a precursor of behavior adoption in the integrated model for predicting health professionals’ behavior [51]. Although intention is not a perfect proxy for future behavior, directly observing peer support behavior among health care workers is neither feasible nor ethical, as these conversations are sensitive and confidential [71]. Using self-reported behavioral intention as a proxy allowed for a practical and more timely evaluation of the CPD course [71]. Self-reported behavior 4 months after course completion was also measured but not used as a primary outcome because there is often a loss to follow-up when measures are not taken soon after course completion [72].

### Data Sources and Measurements

#### Overview

**Figure 2:**
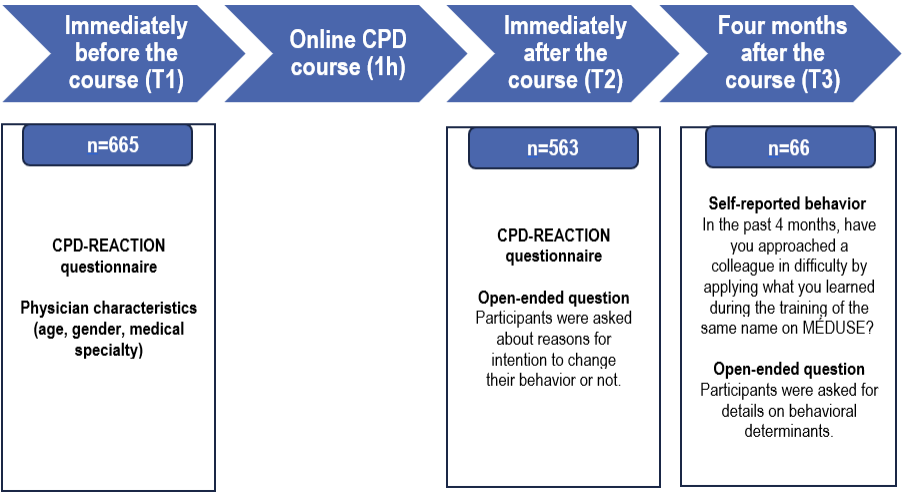
Data sources and collection. CPD: continuing professional development.

The FMSQ collected physicians’ responses on the MÉDUSE online learning platform from March 1, 2022, to June 20, 2024. Data were obtained from three sources: (1) quantitative data, (2) qualitative data, and (3) course content from the MÉDUSE platform (Figure 2). Quantitative data and qualitative data were collected using a questionnaire. The original version administered in French is available in Multimedia Appendix 4 [73,74] and the English translation is available in Multimedia Appendix 5 [73,74]. Physicians completed these online self-administered questionnaires at three timepoints: immediately before the course (T1), immediately after the course (T2), and 4 months after the course (T3) (Figure 2). Questionnaires at T1 and at T2 were administered by a system-generated message displayed at the beginning of the online course, and the questionnaire at T3 was sent automatically by email by the MÉDUSE online platform.

#### Quantitative Data Sources and Measurements

Before the CPD course, we collected data on the age, gender, and medical specialty of participants using an online questionnaire developed by the FMSQ. Before and immediately after the course (T1 and T2), we used the French version of CPD-REACTION to measure physicians’ intention to approach a colleague in difficulty (dependent variable) and the determinants of this intention. Finally, we measured their self-reported behavior of approaching a colleague in difficulty 4 months after course completion (T3).

The CPD-REACTION is a measuring tool whose constructs were developed through a systematic review and whose items were validated using factor analysis [73]. Based on Godin’s integrated model for predicting health professionals’ behavior, the instrument measures intention to adopt a behavior and 4 determinants of intention [53,54,73]. It comprises 12 items grouped into 5 constructs: intention to adopt a behavior (2 items) and four determinants of intention: (1) moral norm (2 items), (2) beliefs about capabilities (3 items), (3) beliefs about consequences (2 items), and (4) social influence (3 items) [73]. Definitions of these determinants are available in Multimedia Appendix 1. Each construct is determined by averaging 2 or 3 items from the instrument [73]. The Cronbach α coefficients for each construct range from 0.79 to 0.89 [73], indicating good reliability [75].

#### Qualitative Data Sources

Overall, participants were asked 8 open-ended questions. Through 2 open-ended questions, physicians were asked about their reasons for intending to adopt the new behavior as well as factors influencing nonadoption of the behavior 4 months later. The remaining 6 questions, which assessed participants’ appreciation of the CPD course, fall outside the scope of this work.

#### Course Content in the MÉDUSE platform

Two reviewers (F Lizotte and EL) were given access to all course content described in the intervention section above. The methodology for the analysis of this data is detailed in the section title “Identifying Behavioral Change Techniques Present in the Course.”

### Data Analysis

For both quantitative and qualitative analyses, we used Godin’s integrated model for predicting health professionals’ behavior as a conceptual framework [51]. In addition, we used Michie’s conceptual framework on BCTs to analyze qualitative responses in detail as well as course content [52]. We analyzed all quantitative data with SAS software (version 9.4; SAS Institute Inc). We performed verification with nonparametric tests using R software (R Foundation for Statistical Computing). The NVivo 15 software (Lumivero) was used for qualitative analyses.

#### Participants’ Intention and Its Determinants to Approach a Colleague in Difficulty, Pre-, and Postcourse

First, we performed descriptive statistics for all variables, including participant characteristics. Then, we calculated the difference between the pre- and postintention mean for each participant using 2-tailed paired *t* tests, assuming normality given our large sample size [76]. To confirm the robustness of the results, we also calculated the statistical significance of intention differences using a nonparametric Wilcoxon signed-rank test [77]. We did the same for each of the 4 determinants of intention to adopt the new behavior (social influences, beliefs about capabilities, beliefs about consequences, and moral norm). Finally, we verified that the results did not differ according to participants’ survey response pattern or participant characteristics (gender, age, and domain of practice). We adopted the age categories used by the Canadian Medical Association in its survey on the wellness of Canadian physicians, as these categories reflect different stages of a medical career [78]. To describe effect size, we calculated Cohen *d*_z_ for within-subjects designs by dividing the mean of the within-person differences with the SD of those differences [79].

#### Estimation of the Effect of the CPD Course on the Intention to Approach a Colleague in Difficulty

We used generalized estimating equations (GEE) with a Gaussian distribution and identity link to estimate the marginal effect of the training program on intention scores over time (pre- vs postcourse [80]). The model included time (pre or post) as the main predictor, with an exchangeable working correlation structure to account for within-individual correlation, and was adjusted for age, sex, domain of practice, and baseline determinants of intention. We included age as a categorical variable since that variable did not respect the postulate of linearity.

#### Predicting Intention Postcourse

To predict intention to approach a colleague in difficulty after the course, we built a multiple linear regression model [81]. Behavioral intention was the dependent variable, while the 4 determinants of this intention and 3 sociodemographic characteristics (age, gender, and domain of practice) were independent variables. A full model including all candidate variables from Godin’s framework was first fitted [51]. Using the backward elimination method, variables with a coefficient *P* value greater than .05 were successively eliminated for a more parsimonious model [82]. Sensitivity analyses were performed at critical α values of .10 or .20 to verify whether different choices changed the predictive performance of our model or the variables included in the final model. We presented the full and the fitted models.

Intention and its determinants were measured as aggregate multiple ordered responses of Likert items from 1 to 7 [73]. Thus, these composite scores approximate continuous latent variables and are commonly analyzed with linear models [83,84]. Simulation and methodological work have shown that treating such multi-item Likert scales (with ≥5 response options) as continuous yields valid estimates and tests in reasonably sized samples [83,84]. We checked for collinearity, normality of residuals, homoscedasticity, and linearity of residuals.

#### Power Analysis

Most participants were already recruited, so the sample size could not be changed. Therefore, we performed a post hoc power analysis. Based on 2 studies examining the effect of CPD courses on physicians’ intentions to adopt new behaviors, we should have been able to detect a mean difference of at least 0.5 in intention before vs after the course [72,85]. The statistical power of our paired *t* test for the primary outcome (mean difference in intention) was 99.9% at a significance level of α=.05 to detect this mean difference of at least 0.5. This indicates that the sample size used in this study was sufficiently large to detect a meaningful effect. We analyzed all quantitative data with SAS software (version 9.4; SAS Institute Inc). We performed verification with nonparametric tests using R software (R Foundation for Statistical Computing).

#### Physicians’ Reasons for Intention to Adopt the New Behavior From Qualitative Data

Through 2 open-ended questions, physicians were asked about reasons for intention to adopt the new behavior as well as factors influencing nonadoption of the behavior 4 months later. The answers were analyzed using a thematic deductive approach [50]. We created an initial codebook by integrating 2 conceptual frameworks: Godin’s integrated model for predicting health professionals’ behavior [51] and Michie’s BCTs with their respective behavioral domains [52]. Two researchers (F Lizotte and EL) organized 10% of the data into themes. Emergent themes were then discussed and added inductively to the framework. We documented these choices in a journal held by F Lizotte. The 2 reviewers (F Lizotte and EL) then coded data independently for greater credibility of the results and compared their analyses [50,86]. During qualitative analysis, F Lizotte, a medical resident, reflected critically about certain themes with EL, who has a background in psychology, and RCC with training in sociology, to improve the trustworthiness of the results. EL was completely blinded to the quantitative analysis process. To obtain consensus on results, any coding differences between the 2 reviewers (F Lizotte and EL) were resolved through discussion with RCC, whose main expertise was qualitative analysis. We described intercoder reliability of thematic analysis of open-ended questions using percentage agreement [87].

#### Triangulating Quantitative and Qualitative Data

We triangulated quantitative and qualitative data to enhance the credibility and validity of the research findings and offer practical theory-based recommendations for CPD course development on peer support [50,88]. We compared 4 determinants of intention, measured with CPD-REACTION and obtained using quantitative methods, with the reasons to adopt the new behavior obtained using qualitative methods [50,88]. We observed where results converged, diverged, or provided additional information [88].

#### Self-Reported Behavior Adoption 4 Months After CPD Course

We calculated the proportion of physicians who self-reported approaching a colleague in difficulty 4 months after the online CPD course. We compared the mean intention of those who adopted the behavior with those who did not using Wilcoxon rank tests because of the small sample sizes of the data at T3 and verified the results using paired *t* tests.

#### Identifying Behavioral Change Techniques Present in the Course

We developed an observation grid (Multimedia Appendix 6 [51,89,90]) from the conceptual framework by Michie et al [52]. Using this grid, 2 independent reviewers (F Lizotte and EL) identified BCTs present in the online CPD course. We analyzed whether the behavioral domains targeted by the BCTs identified in the course were the same as the determinants of intention that had significantly changed after the CPD course. This analysis explored which components of the course might be the active ingredients in changing physicians’ behavioral intentions. Using the entirety of the interrater data, we calculated Cohen kappa to describe interrater reliability for BCT identification [91]. We chose Cohen kappa as the interrater reliability statistic, as it has the advantage of taking into account agreement by chance and can be applied to categorial nominal variables of the observation grid [87].

#### Handling of Missing Data

Item-level missing data were handled by following the CPD-REACTION Questionnaire User Manuel [74]. A nonresponse to 1 of the 2 items measuring a construct of the CPD-REACTION resulted in missing data for the observation of the measured variable [74]. A missing response to an item in the 3-item measure of the determinants of intention was handled with mean imputation [74]. Only 0.25% of responses to items on the CPD-REACTION questionnaire administered at T1 were missing, with item-level missingness ranging from 0.00% to 0.90% (Multimedia Appendix 7). Similarly, approximately 0.27% of responses were missing for items on the CPD-REACTION questionnaire administered at T2, with a range of 0.00% to 1.07% depending on the item (Multimedia Appendix 7). There were no missing sociodemographic characteristics of physicians who completed the CPD-REACTION questionnaire before and after the CPD course.

For wave-level missing data, the 466 participants with data at T1 and T2 were included in the analysis (ie, perfect sets). Sensitivity analyses were conducted to verify if results differed if all data, regardless of data pairing, were analyzed. A complete case analysis was performed in all models.

### Ethical Considerations

We obtained ethical exemption from a hospital research ethics committee (Comité d’éthique de la recherche sectorielle en santé des populations et première ligne—Centre intégré universitaire de santé et de services sociaux de la Capitale-Nationale [CIUSSSCN]; project #2025-3127). The research project participants had given their free and informed consent to take part in the study. Before each questionnaire, a consent form detailed the objective and intended use of the collected data for research (Multimedia Appendix 4 contains the French version and Multimedia Appendix 5 contains the English version). The database was deidentified to protect the confidentiality of participants. A password was required to access the data saved in an Excel file, which has been stored on a secure server. Data analysis was carried out on a CIUSSSCN computer that was also password-protected. Participants experiencing emotional difficulties could speak with peer supporters from the QPHP. Physicians volunteered to participate and received no financial compensation.

## Results

### Participant Flow and Characteristics

**Figure 3:**
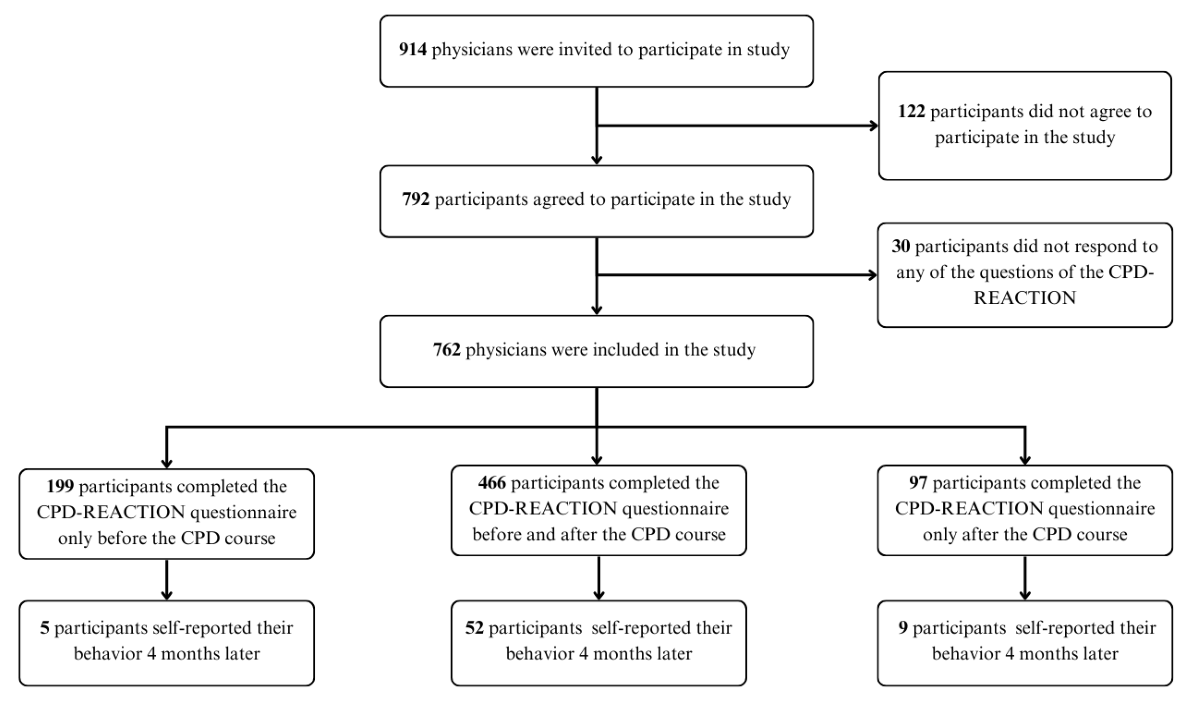
Study flowchart. CPD: continuing professional development.

During the 28-month study period, 914 physicians were invited, of whom 792 (87%) agreed to participate (Figure 3). A total of 30 participants were excluded because they did not answer a single CPD-REACTION questionnaire item. We performed analyses on 61% (466/762) of participants who completed the CPD-REACTION questionnaires before and after the course (T1 and T2). Finally, the frequency of self-reported behavior was calculated on 8% (61/762) of participants who completed both the self-reported behavior change questionnaire 4 months after the course (T3) and the CPD-REACTION questionnaire immediately after the CPD course (T2). We excluded 5 participants who did not complete the postcourse survey (T2) from the analysis of data at T3, as it was only at T2 that we could verify their completion of the course.

Among the participants, the woman-to-man ratio was approximately 1:1, and the majority of physicians (655/762, or 86%) were specialists (Table 1). The average age of participants was 48 (SD 12.4) years, 43.5% (332/762) were women, and 86% (655/762) practiced in a specialty. Physicians who responded both before and after the course (n=466) had similar sociodemographic characteristics. We did not have sociodemographic characteristics for the 97 physicians who responded only to the T2 questionnaire without responding to the T1 questionnaire.

**Table 1:** Characteristics of participants.

Sociodemographic characteristics of physicians				Value (n=762)		Participants who completed the CPD-REACTION questionnaire before and after the CPD^a^ course (n=466)			
**Gender, n (%)**									
	Woman		332 (43.5)		241 (51.7)				
	Man		325 (42.7)		222 (47.6)				
	Other, nonbinary, or prefer not to disclose		8 (1.1)		3 (0.7)				
		Missing		97 (12.7)		0 (0)			
**Domain of practice, n (%)**									
	Surgical specialty		293 (38.5)		208 (44.7)				
	Laboratory specialty		102 (13.4)		71 (15.2)				
	Medical specialty		246 (32.3)		169 (36.3)				
	General practitioner		10 (1.3)		9 (1.9)				
	Nonspecified specialty		14 (1.8)		9 (1.9)				
	Missing		97 (12.7)		0 (0)				
Age (in years), n; mean (SD)				665; 48.1 (12.4)		466; 48.6 (12.3)			

^a^CPD: continuing professional development.

### Participants’ Intention (and its Determinants) to Approach a Colleague in Difficulty, Pre- and Postcourse

Intention means increased from 3.88 (SD 1.73) before to 4.92 (SD 1.40) after the course, as measured on a scale of 1 to 7. This mean difference increase of 1.04 (*P*<.001) is statistically significant (Table 2). This 1-point increase was also clinically significant, with a Cohen *d*_z_ value of 0.72 representing a moderate effect size. Bivariate analyses of changes in the 4 determinants of intention (social influences, beliefs about capabilities, beliefs about consequences, and moral norm) were all statistically significant at an α level of .05. Determinants of intention (beliefs about capabilities, social influences, and beliefs about consequences) increased by statistically significant mean differences ranging from 0.14 to 0.85. Moral norm decreased by a statistically significant mean difference of 0.30 (*P*<.001). Lower extreme values after the CPD course did not explain the decrease in moral norm. The effect size on the increase in beliefs in capacities and on the increase in social influences was moderate. The effect size on the decrease in moral norm was small, and the effect size on the increase in beliefs about consequences was not clinically significant (Table 2). Mean intention and determinants of intention were similar whether participants had responded to both questionnaires (T1 and T2) or to only 1 questionnaire (Table S2 in Multimedia Appendix 8 [73,74]).

**Table 2:** Comparison of behavioral intention before and after the continuing professional development (CPD) course.

CPD-REACTION constructs^a^	Frequency, n	Before CPD course, mean (SD)	After CPD course, mean (SD)	Before vs after CPD course			
				Mean difference (CSD^b^)	95% CI	*P* value^c^	Cohen *d*_z_^d^
Intention	455	3.88 (1.72)	4.92 (1.40)	1.04 (1.45)	0.91 to 1.17	<.001	.72
Beliefs about capabilities	459	4.65 (1.17)	5.49 (0.74)	0.85 (1.11)	0.74 to 0.95	<.001	.76
Moral norm	462	6.09 (1.04)	5.79 (0.77)	–0.30 (1.06)	–0.40 to –0.21	<.001	.29
Social influences	464	3.36 (0.99)	3.81 (0.99)	0.45 (0.74)	0.38 to 0.52	<.001	.61
Beliefs about consequences	465	5.53 (1.04)	5.67 (0.65)	0.14 (0.90)	0.06 to 0.22	<.001	.16

^a^Response scale of the CPD-REACTION questionnaire ranged from 1 to 7 [73,74].

^b^CSD: combined SD.

^c^*P* values were calculated using 2-tailed paired t test.

^d^Cohen *d*_z_ estimates effect size (<0.2=not significant, 0.2-0.5=small, 0.5 to 0.8 medium, >0.8=large) [91].

### Subgroup Analyses of Principal Outcome: Difference of Intention to Approach a Colleague in Difficulty

The increase in the mean difference of intention was approximately 1 point and was statistically significant for all categories of gender, age, and domain of practice (laboratory, surgical, and medical domains) that included at least 10 participants (Multimedia Appendix 9). We did not interpret subcategories containing fewer than 10 participants (eg, general practitioners). Results of the multiple linear regression model confirmed that the intention difference was not modified by gender, age, or domain of practice when all variables were included in the same model (Multimedia Appendix 9).

### Effect of the CPD Course on the Intention to Approach a Colleague in Difficulty

In the GEE model, participation in the CPD course was associated with a higher intention to approach a colleague in difficulty. On average, intention scores were 1.06 points higher in participants after the CPD course compared to before the CPD course (β=1.06; *P*<.001), after adjustment for determinants and for sociodemographic characteristics (Table S1 in Multimedia Appendix 8). Beliefs about capabilities (β=0.37; *P*<.001), social influence (β=0.22; *P*=.002), and beliefs about consequences (β=0.15; *P*=.03) before the CPD course showed significant positive associations with mean intention, whereas moral norm was not significant. No sociodemographic characteristics were independently associated with intention to approach a colleague in difficulty (Table S1 in Multimedia Appendix 8).

### Postcourse Determinants of Intention

In the final multivariate regression analysis model, beliefs about capabilities (β=0.52; *P*<.001), social influences (β=0.27; *P*<.001), and moral norm (β=0.26; *P*<.03) predicted physicians’ intention to approach a colleague in difficulty (Table 3). Our model accounted for 22% of the variance of intention after the course. Sensitivity analyses verified the stability of the other variables and showed consistent results if we also included physicians who had not responded to the precourse questionnaire (Multimedia Appendix 10), except for the variable belief in consequences, whose statistical significance changed when we modelized physicians who had not responded to the precourse questionnaire.

**Table 3:** Predictive factors of physicians’ intention to approach a colleague experiencing difficulties.

Variables^a^		Full multivariate model^b^ (n=457)			Fitted multivariate model^c^ (n=457)		
		β (95% CI)	*P* value		β (95% CI)		*P* value
Beliefs about capabilities		0.39 (0.12 to 0.67)	.005		0.52 (0.28 to 0.75)		<.001
Social influence		0.25 (0.12 to 0.37)	<.001		0.27 (0.15 to 0.39)		<.001
Moral norm		0.26 (0.03 to 0.49)	.02		0.26 (0.03 to 0.48)		.03
Beliefs about consequences		0.22 (–0.01 to 0.45)	.06		N/A^d^		N/A
**Age (in years; reference: 35-54)**				.39^e^			
	<35	–0.22 (–0.56 to 0.13)	.21		N/A		N/A
	>54	–0.11 (–0.37 to 0.14)	.38		N/A		N/A
**Domain (reference: surgical)**				.25^e^			
	Laboratory	0.23 (–0.12 to 0.57)	.20		N/A		N/A
	Medical	0.29 (0.03 to 0.55)	.03		N/A		N/A
	Family medicine	0.39 (–0.45 to 1.23)	.36		N/A		N/A
	Other	0.30 (–0.54 to 1.13)	.48		N/A		N/A
**Gender (reference: women)**				.54^e^			
	Men	–0.12 (–0.37 to 0.12)	.33		N/A		N/A
	Other, nonbinary, or prefer not to disclose	–0.44 (–1.86 to 0.97)	.54		N/A		N/A

^a^We included psychosocial variables measured after the course.

^b^*R*^2^=0.24.

^c^*R*^2^=0.22.

^d^N/A: not applicable, as variables with a coefficient *P* value greater than .05 were successively removed using the backward elimination method.

^e^Global *P* value.

Postulates of independence of observations and homoscedasticity were respected. Postulates of normality and linearity of residuals were not respected. We verified the correlations between the independent variables and found they did not explain our model’s low predictive capacity (Multimedia Appendix 10).

### Physicians’ Reasons for Intention to Adopt the New Behavior From Qualitative Data

Of the 251 open-ended responses related to behavioral intentions (T2), 153 verbatims expressed clear behavioral intentions (eg, “I will help my peer”), 10 showed hesitation (eg, “I will try to”), 89 described reasons for intention to approach a colleague in difficulty (eg, “I realize more fully my ability to act”), and 8 referred to other related behaviors. The above categories are not mutually exclusive. Physicians most frequently intended to (1) be more attentive to their colleagues’ signs of distress, (2) approach a colleague with kindness or help or support a colleague, and (3) be more available or listen more to colleagues (Multimedia Appendix 11). The reasons for intention to change behavior physicians most frequently cited were beliefs about capabilities, beliefs about consequences, and knowledge of the behavior (Table 4). For each of the 10 categories of reasons for wanting to adopt the new behavior, intercoder percentage agreement on thematic analysis varied between 98.1% and 99.5%.

**Table 4:** Reasons for intention to adopt new behavior of approaching a colleague in difficulty.

Reasons for intention to adopt new behavior	Representative excerpts of verbatim	Agreement, n (%)
Beliefs about capabilities	“I realize more fully my ability to act.” “I have more tools to approach a colleague in difficulty.” “I feel more comfortable doing it.”	41 (98.4)
Beliefs about consequences	“This limits the consequences for the colleague and the team.” “To improve the collegiality of my department.” “I think that the current context of healthcare depends on the support we provide to our colleagues, so it is essential to do it.”	16 (99.2)
Knowledge	“Better knowledge of how to detect a colleague's distress and how to intervene in such cases.” “Better understanding of the approach.” “I will better assess where my colleague stands on the distress scale.”	11 (96)
Social/professional role and identity	“Necessary with my hospital duties.” “Feeling more legitimized to approach a colleague in difficulty.” “Better understanding and responsibility of collegiality.”	6 (98.5)
Moral norm	“I like the idea that doing nothing is not an option.” “Not acting is not an option.” “…understanding that some approaches may be better than others, but anything is better than doing nothing.”	5 (97.9)
Past behavior or habit related to behavior	“It has provided me with new tools to continue supporting colleagues in difficulty.” “I already apply many similar principles.” “Continue to approach my colleague when they seem to be having difficulties.”	4 (99)
Emotion related to behavior	“Being more willing to get uncomfortable.” “It reassures me.” “I feel more equipped and reassured about my reflections.”	3 (99.5)
Environmental context and resources	“Colleague to help.” “Frequent problem.”	2 (98.9)
Action planning	“Do not procrastinate.” “Do not hesitate to create opportunities outside of work.”	2 (98.1)
Memory, attention, and decision processes	“Because I realize that often, I get too absorbed in my professional or personal concerns to look around me. Being a witness allows for perspective and a new outlook.”	1 (99)

The reasons physicians gave for not wanting to change their practice were mostly related to their past behavior, for example, that they were already helping a peer and thus had already adopted the behavior (Multimedia Appendix 11). Other reasons for not intending to change behavior were their environmental context (eg, “I’m now in solo practice”) and that helping a peer was not relevant to their particular medical practice. Four months after the CPD course, the behavioral determinants most frequently given for not approaching a colleague in difficulty were having had no occasion to adopt the behavior, having no colleagues in difficulty, and not being actively in practice or not being in full-time practice (Multimedia Appendix 11).

### Triangulated Quantitative and Qualitative Data

Qualitative and quantitative results showed most physicians were motivated to approach a colleague in difficulty following the online CPD course. Both qualitative and quantitative results pointed to beliefs about capabilities as being an important determinant of intention. Beliefs about consequences was an important theme in the qualitative data, but it was not an important determinant of postcourse intention to approach a colleague in difficulty. Sensitivity analysis of the 251 physicians who provided open-ended responses related to behavioral intentions (T2) also showed a divergence regarding beliefs in consequences between qualitative and quantitative results.

### Self-Reported Behavior Adoption 4 Months After CPD Course

Among the 66 physicians who completed the self-administered questionnaire at T3, 61 had completed CPD-REACTION immediately after the course. Of these, 41% (25/61; 95% CI 28.6%-54.3%) reported approaching a colleague in difficulty in the 4 months following the CPD course. Those who adopted the behavior had a mean intention after the CPD course of 5.40 (SD 1.01), and those who did not adopt the behavior had a mean intention of 5.00 (SD 1.40). This 0.4 (SD 1.26; *P*=.24) mean behavioral intention difference is not statistically significant.

### Behavioral Change Techniques Identified as Present in the Course

A total of 7 BCTs were found in the online CPD course: goal setting, increasing competence, planning, persuasive communication, behavior-related information, modeling, and behavioral experiments (Table 5). Cohen kappa measuring interrater reliability was 0.53 (95% CI 0.14-0.91) for evaluation of BCTs identified the course, which represents a moderate strength of agreement [92]. Of these 7 BCTs, 4 were related to behavioral intentions, 3 involved competence, 2 involved behavioral regulation, and 2 were related to beliefs about consequences (Multimedia Appendix 11). Beliefs about capabilities, knowledge, social influences, and memory, attention, and decision processes were each targeted by 1 BCT.

**Table 5:** Behavioral change techniques (BCTs) identified in the continuing professional development (CPD) course.

BCT	Definition	Domains targeted
Goal setting	Clearly indicate the targeted behavior.	Competence, behavioral intention, behavioral regulation
Increasing competence	Enhance skills through problem-solving, decision-making, and goal setting.	Competence, beliefs about capabilities, behavioral intention
Action planning	Identify the components of the behavior and establish a plan to execute each component, including when and where the behavior will be performed (ie, scheduling behaviors).	Memory, attention, and decision processes, behavioral regulation
Persuasive communication	A credible source presents arguments in favor of the behavior. There must be a presentation of arguments; general pro-behavior communication does not qualify.	Beliefs about consequences, behavioral intention
Behavior-related information	Provide information about the antecedents or consequences of the behavior, the connections between them, or relevant BCTs.	Knowledge, beliefs about consequences, behavioral intention
Modeling	Observe the behavior of others as a model for action.	Competence, social influences
Behavioral experiments	Ask individuals to test hypotheses about the behavior, its causes, and its consequences by collecting and interpreting data, including personal experiences.	No consensus on targeted domains

## Discussion

### Principal Findings

We assessed the effect of an online CPD course on physicians’ intention to approach a colleague in difficulty and analyzed why participants considered integrating this peer support approach or not into their practice. Mean behavioral intention increased significantly after the course. Postcourse, significant determinants of intention were social influences, beliefs about capabilities, and moral norm. Most frequently stated reasons for intention to change behavior were beliefs about capabilities, beliefs about consequences, and knowledge of behavior. Triangulation showed an alignment of quantitative and qualitative results on beliefs about capabilities as a key factor but diverged on beliefs about consequences. In the 4 months following the CPD course, 41% (25/61) of physicians reported approaching a colleague in difficulty. There was no significant difference in the intention to approach a colleague in difficulty between physicians who adopted this behavior at the 4-month follow-up and those who did not. Those who did adopt the behavior had a nonstatistically significant higher intention after the CPD course than those who did not. Finally, we identified 7 BCTs in the online CPD course: goal setting, increasing competence, planning, persuasive communication, behavior-related information, modeling, and behavioral experiments. These results lead us to make the following observations.

### Comparison of Participants’ Behavioral Intention Pre- and Postcourse With Prior Work

First, we found that, on average, physicians reported a stronger intention to approach a colleague in difficulty after completing the CPD course compared to before. This 1-point intention increase was statistically significant and also clinically significant, having a moderate effect size. This increase was observed in bivariate analysis and confirmed with multivariate analysis. This suggests that the CPD course motivated physicians to approach colleagues in difficulty. Enhancing peer support among physicians through CPD courses is particularly important as it can play a protective role regarding psychological distress in physicians [17,18].

Also, to the best of our knowledge, this 1-point intention increase represents a greater increase in intention than has been observed in most other studies using the CPD-REACTION to evaluate changes in health professionals’ intentions following an intervention [72,85,93-102]. The before-after difference of intention was more than double the difference observed in a study by Ayivi-Vinz et al [85] who evaluated intention before and after 9 online CPD courses taken by Quebec physicians, all on topics other than this one. The 95% CIs of difference in intention between their study and ours do not overlap [85]. Thus, the observed difference between the 2 studies is statistically significant. An important difference is that Godin’s integrated model for predicting health professionals’ behavior was used by the senior instructional designer (MT) to design the course, while the 9 courses in the study by Ayivi-Vinz et al [85] were developed by physicians who may not have used behavior change models in course design. This may give support to the importance of using theory to produce CPD courses. Another explanation is that preintervention intention among the Quebec physicians in the study by Ayivi-Vinz et al [85] was already high at 5.5 out of 7, while in ours it was 3.9 out of 7, and thus there was more room for improvement. Overall, this result suggests that there are benefits of using behavior change theories, especially for CPD courses targeting behaviors that physicians are initially less inclined to adopt. It also suggests that it is possible to increase behavioral intention in physicians using online training formats.

### Change in Determinants of the Intention to Approach a Colleague in Difficulty

Social influences, beliefs about capabilities, and beliefs about consequences increased. An unexpected postcourse decrease in moral norm occurred. Moral norm had the highest mean among the 4 determinants of intention both before and after the intervention, indicating that participants already felt a moral obligation to adopt the targeted behavior and thus there was little room for improvement. Also, the apparent decrease had a small effect size, which may not translate into clinical significance. One possible explanation for this unexpected decrease in moral norm is that participants may have given more nuanced answers after the CPD course as they better understood the ethical and deontological implications of approaching a colleague in difficulty. In the precourse questionnaire, 38.3% (177/462) of the physicians answered with the maximum score of 7/7 for moral norm, compared to none after the intervention. Nevertheless, further qualitative data analysis is needed to explore this result. Analyzing which BCTs target moral norm could also be important, as this is not studied by Michie et al [52].

### No Modifying Effect of Age, Gender, or Domain of Practice on Primary Outcome

Second, we also examined whether participant characteristics modified the impact of the course on their behavioral intention. The before–after intention difference was the same regardless of age, gender, and domain of practice (laboratory, surgical, and medical domains). Thus, the course successfully reached physicians regardless of their sociodemographic characteristics and does not need to be adapted for a specific population. For example, a previous study found that surgeons were the least likely physicians to use peer support [34], hence the importance of our finding that surgeons also responded favorably to the online CPD course.

### Comparison of Determinants of Intention Pre- and Postcourse With Prior Work

Third, we found that determinants of intention to approach a colleague in difficulty after the CPD course included social influences, beliefs about capabilities, and moral norm. Beliefs about capabilities was the strongest predictive variable of the intention to approach a colleague in difficulty. This means improving physicians’ perception of their self-efficacy in adopting a behavior, as well as their perceived control over performing that behavior, is important to motivate them to approach a colleague in difficulty. Thus, CPD course developers should target beliefs about capabilities through BCTs for peer support training. Beliefs about capabilities was also the most significant determinant of intention in Godin’s systematic review on health care workers’ behaviors [53]. BCTs that are known to be effective for this include: (1) increasing skills, (2) coping strategies, (3) repetition or practice of the behavior, (4) feedback, (5) planned self-affirmations, (6) motivational interviewing, and (7) graded tasks (starting with tasks that are easy to accomplish and gradually making them more difficult). In previous studies, beliefs about capabilities, moral norm, and beliefs about consequences were all predictive of intention to adopt behaviors related to patient care (eg, “Prescribe opioids in a safe manner when managing perioperative pain in my patients”) postcourse [72,85]. Our results differ slightly from these, with social influence also being predictive of this intention. Approaching a colleague in difficulty is a social behavior that can be adopted by a whole departmental team [38]. Our results suggest that the social context may be a more important influence on team-based behaviors than on clinical behaviors, which are associated more with individual clinicians.

### Comparing Predictive Capacity Postcourse With Prior Work

Our model had a low predictive capacity (*R*²=0.22), explaining only 22% of the variance in intention. In comparison, previous literature showed that the predictive capacity of models analyzing 1 single behavior varied between 19% and 62%, depending on the type of behavior [53]. Bakwa et al’s [72] study of multiple behaviors related to patient care among Quebec physicians explained 82% of the variance in physicians’ intention to adopt the clinical behavior. However, Bakwa et al [72] included multiple behaviors in the same model, while we studied only 1 behavior, and moreover one that is specific to the well-being of physicians rather than geared to a clinical task. Indeed, existing studies mainly predict behaviors related to patient care [53,72]. While variables of Godin’s integrated model for predicting health professionals’ behavior are sufficient to predict such behaviors [51], approaching a colleague is not a clinical behavior and may be influenced by factors not measured by Godin’s model such as lack of time, lack of privacy, and organizational culture [36,103]. This is significant because the Quintuple Aim—adopted by many health care systems—now emphasizes 5 key outcomes, and not all of them clinical: patient outcomes, patient experience, efficiency, clinician well-being, and equity [13]. Therefore, further development is needed regarding fostering behaviors related to clinician well-being.

### Triangulated Quantitative and Qualitative Data

Fourth, triangulating quantitative with qualitative results confirmed that beliefs about capabilities was a key determinant of intention to approach a colleague in difficulty. Interestingly, results regarding beliefs about consequences diverged slightly: while it was the second most frequent reason for intention to change behavior stated in physicians’ open-ended responses, it was not a statistically significant determinant of intention in our quantitative results. One explanation is that statistical significance varied according to the other variables included in the model, perhaps because the *P* value for beliefs about consequences was so close to the critical α value of .05. Another possible explanation is that beliefs about consequences, as described by Godin [51], seems to refer to the consequences for oneself. Items for measuring beliefs about consequences quantitatively were worded as follows “Overall, I think that *for me* approaching a colleague in difficulty would be....” [73]. In contrast, in our qualitative analysis, beliefs about the consequences of adopting the behavior could also refer to its consequences for the colleague being approached, the team, the department, or for society (Table 5). Indeed, many physicians taking part in this study perceived that the consequences of approaching a colleague in difficulty would be beneficial for the team and the department, a theme often mentioned in the open-ended responses. Thus the perception of the consequences of team-focused care may include organizational benefits, team benefits, patient benefits, and benefits to individual team members [104]. Also, approaching a colleague in difficulty is a team-focused behavior that takes place between 2 health care workers, while behaviors related to patient care occur between a clinician and a patient (eg, prescribing medication). We hypothesize that such team-focused behaviors may depend on a wider variety of determinants, such as organizational culture, than do individual behaviors related to patient care [36,104,105]. Whether peer support strategies can be applied without fear of judgment or retaliation within the organization may depend on its climate of psychosocial safety, or the perception among employees that their psychological health is a priority for the management [106,107]. A study by Tolin et al [38] reported that physicians’ perception of institutional support had a positive impact on implementation of a physician-focused peer support program. Broadening the item statements on beliefs about consequences to include effects on others and adding questions with regards to role and identity may be a good starting point for improving measuring tools regarding team-based behaviors.

Other determinants to measure in future studies should include social and professional role or identity and past behavior or habits related to the behavior, both mentioned by participants as determinants of intention and present in Godin’s integrated model for predicting health professionals’ behavior (Table 4) [51]. Our qualitative analysis identified the following further potential determinants: (1) knowledge; (2) action planning; (3) memory, attention, and decision processes; (4) environmental context and resources; and (5) emotion related to behavior. The first 3 of these determinants are also behavioral domains targeted by the BCTs we identified in the CPD course.

### Self-Reported Behavior Adoption 4 Months After CPD Course

Fifth, 4 of 10 physicians reported having approached a colleague in difficulty in the 4 months following the CPD course, and those who did so had a higher intention to adopt the behavior after the CPD course. No statistically significant difference in intention to approach a colleague in difficulty was found between those who did and those who did not adopt the behavior (5.4 vs 5.0; *P*=.24). This unexpected finding is possibly due to the limited statistical power of the analysis, estimated at 23%. Also, environmental factors, such as practicing solo, were the barriers most mentioned by physicians. Some of these physicians who might have been willing to approach a colleague in difficulty were simply not in the right environment to be able to do so. Other studies report on peer support courses that are part of comprehensive peer support programs that include an organization-wide referral system [15,22,26,27,30,34,38-40,43]. Thus, a physician could be the only physician in a department and still participate in a peer support program thanks to the referral system. It would be valuable to further evaluate whether formalizing peer support through these programs helps overcome certain environmental barriers or, conversely, introduces new barriers, compared to informal peer support outside of a program, as occurred in our study.

### Behavioral Change Techniques Present in the Course

Lastly, we identified 7 BCTs in the online CPD course. This is the first time that BCTs have been identified in an FMSQ course and triangulated with before-and-after measures of CPD-REACTION. The results suggest that the BCTs effectively targeted determinants of intention, such as beliefs about capabilities and social influence, which had changed after the course. However, this study, which analyzed only 1 course, cannot make recommendations on which BCTs to use for which behaviors. Nevertheless, our results offer significant methodological progress in evaluating CPD course content, which could lead to innovations in CPD course development. Further studies on the use of theory-informed BCTs in CPD course design to enhance clinician adoption of targeted behaviors could further our understanding of effective course development strategies.

### Limitations

First, the quasi-experimental before-and-after study design is vulnerable to confounding concomitant events that could influence study outcomes because the design comprises only a single group [50]. However, the short duration of the online CPD course (1 hour) made it less likely for other events to introduce bias. We chose this before-and-after study design as it is better suited to the constraints of practice settings than randomized control trials and better reflects the real-world CPD context [50]. Furthermore, we confirmed bivariate analysis with the GEE model to better measure the effect of the CPD course. Triangulation of quantitative with qualitative methods improved the internal validity of the study [50,108].

Second, our results mainly apply to specialist physicians who chose to follow a nonmandatory CPD course and thus had some interest in the subject. The effect of the CPD course could have differed if it were mandatory for all physicians, as seen in other education programs [109]. Furthermore, general practitioners were underrepresented in this study (n=9). Thus, we do not know how general practitioners would respond to the CPD course but have no reason to think that they would behave differently than specialists. In addition, the well-being of all health care professionals is paramount, and it is not clear if the online CPD course would have any impact on other types of health professionals.

Third, there was a possible social desirability bias whereby physicians overrated their intention to approach a colleague in difficulty [50]. Confidentiality and anonymity were ensured to limit this bias. Of note, we measured intention using the same instrument used by Bakwa et al [72] and by Ayivi-Vinz et al [85] in their studies, so the desirability bias would have applied to their results as well and does not explain the higher before-and-after intention difference we observed in this study.

Fourth, this study does not allow us to confirm the reasons for which results regarding beliefs about consequences diverged, reasons to change behavior that were prominent in qualitative results but unstable or nonsignificant in the multiple linear regression. Although hypotheses are detailed in the Discussion, further studies would be needed to validate them.

Fifth, another limitation is that we used a backward elimination procedure, which can increase the risk of overfitting and biased effect estimates. However, results in the full model and fitted model were similar.

Sixth, we lacked statistical power to measure the difference in intention to approach a colleague in difficulty between those who did and those who did not adopt the behavior.

Lastly, self-reporting of behavior may also overestimate behavior adoption, as participants may not have used the approach taught in the course and may still report having adopted the behavior. Furthermore, only a small proportion of participants completed the self-reported behavior questionnaire 4 months after the course. This may indicate a selection bias in favor of the most motivated physicians, which could overestimate the proportion of those adopting the behavior. The sensitivity analysis in Multimedia Appendix 8 shows that this potential selection bias on number of questionnaires answered does not affect the primary outcome (mean difference in intention to approach a colleague in difficulty) but only the secondary outcome of self-reported behavior 4 months after the training.

### Conclusions

To the best of our knowledge, this is the first study that uses validated before-and-after intervention measures to examine the effect of a CPD course on physicians’ intention to approach a colleague in difficulty (experiencing distress, burnout, or second-victim symptoms after a patient adverse event). The CPD course produced an increase in physicians’ intention regardless of age, gender, or domain of practice (laboratory, surgical, and medical domains). Beliefs about capabilities was an important determinant of intention to approach a colleague in difficulty. Although the CPD course increased physicians’ intention to approach a colleague in difficulty, there is still a gap in understanding what other factors predict this intention. Within 4 months of the CPD course, 41% (25/61) of the physicians reported approaching a colleague in difficulty using strategies taught in the online CPD course. Identifying BCTs in the CPD course design was a methodological innovation for FMSQ course evaluation.

Although the CPD course demonstrated a positive effect on physicians’ intention to approach a colleague in difficulty, more research is needed to better understand the mechanisms through which the intervention works. This could be done by translating the course into English, making it available to English-speaking physicians in Quebec and other Canadian provinces or internationally, and measuring the effect of the CPD course on approaching a colleague in difficulty in other contexts. Furthermore, instructional designers should focus on enhancing physicians’ beliefs about their capabilities to facilitate their adoption of this peer support behavior. Instructional designers could consider using BCTs, especially when targeted behaviors are less widely accepted by clinicians or perceived as more difficult to adopt. Evaluating the effect of BCTs on behavioral intention could deepen our understanding of interventions aimed at changing clinicians’ behaviors.

Our results suggest that peer support courses help build a culture of care among physicians. By enhancing our knowledge of interventions aimed at improving the peer support capabilities of physicians, this project indirectly aimed to improve the well-being of health care professionals. Peer-supported professionals could then be better able to contribute to resilient health care systems and to providing quality care to patients.

## Appendix

Multimedia Appendix 1 Conceptual framework.

Multimedia Appendix 2 Strengthening the Reporting of Observational Studies in Epidemiology (STROBE) Checklist.

Multimedia Appendix 3 Standards for Reporting Qualitative Research (SRQR) Checklist.

Multimedia Appendix 4 Complete Questionnaires and Consent Forms (French version).

Multimedia Appendix 5 Complete Questionnaires and Consent Forms (English version).

Multimedia Appendix 6 Grid for evaluating the CPD course "Approaching a Colleague in Difficulty" offered by the Fédération des médecins spécialistes du Québec (FMSQ) according to Michie’s Behavior Change Techniques.

Multimedia Appendix 7 Number and percentage of participants with missing data for each variable.

Multimedia Appendix 8 Assessing Robustness in Bivariate Comparisons.

Multimedia Appendix 9 Analysis of mean intention difference by age, gender and by domain of medical specialty.

Multimedia Appendix 10 Sensitivity analysis.

Multimedia Appendix 11 Supplementary qualitative results.

## Abbreviations

BCT: behavior change technique
CIUSSSCN: Centre intégré universitaire de santé et de services sociaux de la Capitale-Nationale
CPD: continuing professional development
FMSQ: Fédération des médecins spécialistes du Québec
GEE: generalized estimating equations
QPHP: Québec Physicians’ Health Program
SRQR: Standards for Reporting Qualitative Research
STROBE: Strengthening the Reporting of Observational Studies in Epidemiology
TIDieR: Template for Intervention Description and Replication

## Data Availability

The datasets generated or analyzed during this study are available from the corresponding author on reasonable request.
