# Supplementary material for: Effect of an Online Continuing Professional Development Course on Physicians’ Intention to Approach a Colleague in Difficulty: Mixed Methods Convergent Study
Source: JMIR Med Educ. 2026 Feb 5;12:e80199. doi: 10.2196/80199 (PMC12921432; doi:10.2196/80199)
Supplement: Multimedia Appendix 1 [file mededu_v12i1e80199_app1.docx]

**Multimedia Appendix 1:** **Conceptual framework**

Two conceptual frameworks that can be useful in examining the effect of CPD courses on behavior are Godin’s integrated model for predicting health professionals’ behavior [1], and Michie's Behavior Change Techniques [2]. As shown in Figure 1, these frameworks share the concept that behavioral determinants lead to behavior adoption [1-3] and the frameworks have many behavioral determinants in common [1-3]. Both models share behavioral determinants such as intention, beliefs about capabilities, beliefs about consequences and social influences as shown in Figure 1 [1-3]. These shared concepts are depicted by dotted lines in Figure 1. Michie et al. also propose that behavior change techniques (BCTs) can influence and target behavioral determinants which in turn translate into behavior change [2]. In Figure 1, constructs marked with “a” are determinants which can be targeted by BCTs.


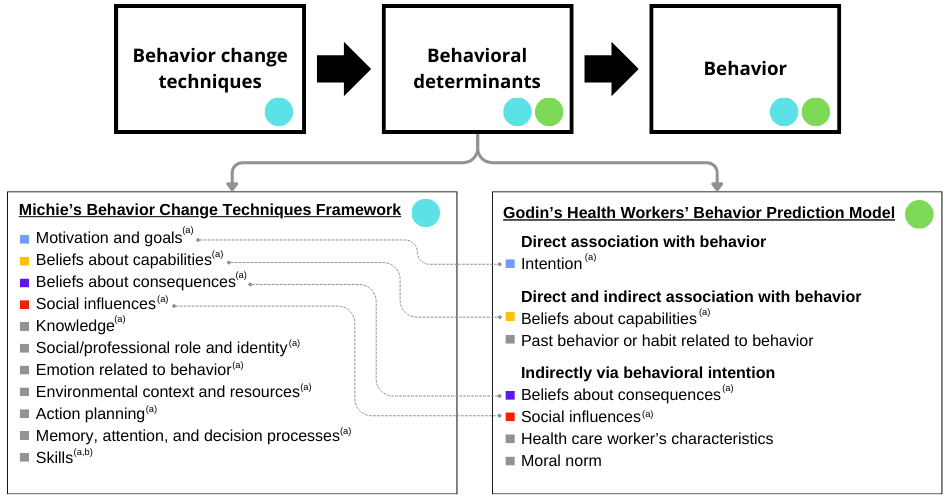


Figure 1. Framework combining Godin’s integrated model for predicting health professionals’ behavior with Michie's Behavior Change Techniques Framework [2, 3]

Note: Constructs marked with “a” in the figure are determinants which can be targeted by BCTs, while skills (marked “b”) were not measured as physicians’ behavior was not directly observed.

According to Michie et al., 137 BCTs can be incorporated into an intervention to facilitate behavior change by addressing one or more behavioral determinants. Michie et al. name these determinants « behavior domains » in their article [2]. One example of a BCT is planning, which consists of inviting participants to plan a behavior [2]. The planning BCT targets four behavioral determinants: memory, attention, decision processes, and behavioral regulation [2].

Godin’s integrated model for predicting health professionals’ behavior, developed using factor analysis, specifies whether behavioral determinants are directly or indirectly linked to behavior [1, 3]. Direct determinants of behavior are intention, beliefs about capabilities and past behavior (Figure 1) [1, 3]. Beliefs about consequences, social influences, moral norm and healthcare workers’ characteristics as well as beliefs about capabilities and past behavior are indirectly linked to behavior through intention [1, 3].

**Definitions of Behavioral Determinants**

Beliefs about capabilities
“Beliefs about capabilities” refer to an individual’s perception of their self-efficacy in adopting a behavior, as well as their perceived control over performing that behavior, even in the presence of barriers [1, 4]

Beliefs about consequences
“Beliefs about consequences” refer to the healthcare professional’s perceptions regarding the outcomes or consequences of adopting or not adopting the behavior [1, 4]

Moral norm
The moral norm refers to beliefs about the moral obligation to adopt the targeted behavior [1, 4]. Based on their personal values and principles, a healthcare professional will feel a strong or weak personal obligation to adopt the targeted behavior.

Social influences
Social influence refers to the healthcare professional’s perception of the opinions of significant others regarding the adoption of the targeted behavior [1, 4].

Behavioral intention or motivation
This term refers to the intention or willingness to adopt the targeted behavior [1, 2].

Social/professional role and identity
Social/professional role and identity refer to how the professional perceives the expected behavior based on their identity (e.g., age, gender, social status, or professional role) [1].

Past behavior / habit
Past behavior or habit is defined as the frequency with which the behavior has been performed in the past by the healthcare professional, which may include a degree of automatization [1, 4].

Knowledge
This term encompasses knowledge about the health condition related to the behavior, the scientific rationale for adopting the behavior, and the procedural knowledge of how the behavior is carried out by the healthcare professional [2].

Memory, attention, and decision-making processes
These terms refer to interconnected cognitive functions that allow one to remember to perform the behavior or to pay attention to performing the behavior [2].

Environmental context and resources
Environmental context and resources refer to the physical, material, and temporal factors that may facilitate or hinder the adoption of a behavior [2].

Behavioral regulation
This term refers to the actions of setting one or more goals related to the targeted behavior, prioritizing these goals, and planning the actions needed to achieve them [2].

Emotion
This category refers to emotional factors that facilitate or hinder the adoption of the behavior, including affect, stress, anticipated regret, fear, burnout, cognitive overload, fatigue, anxiety, and depression [2].

Références

1. Godin G. Les comportements dans le domaine de la santé : comprendre pour mieux intervenir. Montréal: Presses de l'Université de Montréal; 2012. ISBN: 9782760627796 2760627799.

2. Michie S, Johnston M, Francis J, Hardeman W, Eccles M. From Theory to Intervention: Mapping Theoretically Derived Behavioural Determinants to Behaviour Change Techniques. Applied Psychology. 2008;57(4):660-80. doi: 10.1111/j.1464-0597.2008.00341.x.

3. Godin G, Bélanger-Gravel A, Eccles M, Grimshaw J. Healthcare professionals' intentions and behaviours: A systematic review of studies based on social cognitive theories. Implementation Science : IS. 2008;3:36. doi: 10.1186/1748-5908-3-36.

4. Légaré F, Borduas F, Freitas A, Jacques A, Godin G, Luconi F, Grimshaw J. Development of a simple 12-item theory-based instrument to assess the impact of continuing professional development on clinical behavioral intentions. PLoS One. 2014;9(3):e91013. PMID: 24643173. doi: 10.1371/journal.pone.0091013.
