# Supplementary material for: Effect of an Online Continuing Professional Development Course on Physicians’ Intention to Approach a Colleague in Difficulty: Mixed Methods Convergent Study
Source: JMIR Med Educ. 2026 Feb 5;12:e80199. doi: 10.2196/80199 (PMC12921432; doi:10.2196/80199)
Supplement: Multimedia Appendix 2 [file mededu_v12i1e80199_app2.docx]

**Multimedia Appendix 2: Strengthening the Reporting of Observational Studies in Epidemiology (STROBE) Checklist**

| Section | Item No. | Recommendation | Check |
| --- | --- | --- | --- |
| TITLE and ABSTRACT | 1 | (a) Indicate the study’s design with a commonly used term in the title or the abstract. (b) Provide in the abstract an informative and balanced summary of what was done and what was found. | Page 1, L1-2  Pages 2-3, L36-71 |
| INTRODUCTION | | |  |
| Background/rationale | 2 | Explain the scientific background and rationale for the investigation being reported. | Pages 4-5, L103-136 |
| Objectives | 3 | State specific objectives, including any prespecified hypotheses. | Page 5, L137-140 |
| METHODS | | |  |
| Study design | 4 | Present key elements of study design early in the paper. | Page 5, Line 144-154 |
| Setting | 5 | Describe the setting, locations, and relevant dates, including periods of recruitment, exposure, follow-up, and data collection. | Page 6, L167-172  Pages 6-7 L179-208 |
| Participants | 6 | (a) Cohort study—Give eligibility criteria and sources/methods of selection of participants. Describe methods of follow-up. (b) Case-control study—Give eligibility criteria and sources/methods of case ascertainment and control selection. Give rationale for choice of cases and controls. (c) Cross-sectional study—Give eligibility criteria and sources/methods of selection of participants. | Page 6, L167-173 |
| Variables | 7 | Clearly define all outcomes, exposures, predictors, potential confounders, and effect modifiers. Give diagnostic criteria, if applicable. | Main outcome: Page 7, L211-220  Predictors: Page 8, L240-244 and Multimedia Appendix 1 |
| Data sources/measurement | 8* | For each variable of interest, give sources of data and details of methods of assessment (measurement). Describe comparability of assessment methods if more than one group. | Page 8-9, L223-257 |
| Bias | 9 | Describe any efforts to address potential sources of bias. | Confounding concomitant events, Page 24 L657-663  Social desirability, Page 25, L672-676  Selection biais, Page 24 L713-720  Potential biais induced by Handling of missing data: Page 12, L356-368 and sensitivity analysis in Multimedia Appendix 7 and 9 |
| Study size | 10 | Explain how the study size was arrived at. | Page 6, L167-168 and L173-175 |
| Quantitative variables | 11 | Explain how quantitative variables were handled in the analyses. If applicable, describe which groupings were chosen and why. | General variable handling: Page 9-10, L272-308 |
| Statistical methods | 12 | (a) Describe all statistical methods, including those used to control for confounding. (b) Describe any methods used to examine subgroups and interactions. (c) Explain how missing data were addressed. (d) Cohort study—Explain how loss to follow-up was addressed. (e) Case-control study—Explain how matching of cases and controls was addressed. (f) Cross-sectional study—Describe analytical methods accounting for sampling strategy. (g) Describe any sensitivity analyses. | Pages 9-12, L272-368 |
| RESULTS | | |  |
| Participants | 13* | 1. Report numbers of individuals at each stage of the study. 2. Give reasons for non-participation at each stage. 3. Consider use of a flow diagram. | Page 12-13, L381-398  Page 13, Figure 3 |
| Descriptive data | 14* | (a) Give characteristics of study participants (e.g., demographic, clinical, social) and information on exposures and potential confounders. (b) Indicate number of participants with missing data for each variable. (c) Cohort study—Summarize follow-up time (e.g., average and total amount). | Page 13-14, Table 1  Page 11-12,  Page13, L382-389; L394-398 and Multimedia Appendix 7 |
| Outcome data | 15* | Cohort study—Report numbers of outcome events or summary measures over time. Case-control study—Report numbers in each exposure category or summary measures of exposure. Cross-sectional study—Report numbers of outcome events or summary measures. | Page 13, L402-414  Page 14-15, Table 2 |
| Main results | 16 | (a) Give unadjusted and, if applicable, adjusted estimates with precision (e.g., 95% CI). State confounders adjusted for and why. (b) Report category boundaries when continuous variables were categorized. (c) If relevant, translate estimates of relative risk into absolute risk for a meaningful time period. | Unadjusted outcome: Page 14-15, Table 2  Adjusted estimates of main outcome: Page 16, Table 3 |
| Other analyses | 17 | Report other analyses done—e.g., subgroup analyses, interactions, sensitivity analyses. | Subgroup analyses: Page 15, L424-430 and Multimedia Appendix 10  Sensitivity analyses, Multimedia Appendix 8 |
| DISCUSSION | | |  |
| Key results | 18 | Summarize key results with reference to study objectives. | Page 20, L510-522 |
| Limitations | 19 | Discuss limitations, considering potential bias or imprecision. Discuss both direction and magnitude of any potential bias. | Page 24, L657-694 |
| Interpretation | 20 | Provide a cautious interpretation considering objectives, limitations, multiplicity of analyses, results from similar studies, and other evidence. | Pages 20-24, L524-654  Pages 25-26 L 697-719 |
| Generalizability | 21 | Discuss generalizability (external validity) of the study results. | External validity, Page 25, L665-670 |
| OTHER INFORMATION | | |  |
| Funding | 22 | Give source of funding and role of funders for the present study and, if applicable, for the original study. | Page 27, L759-760 |

Source: Vandenbroucke JP, von Elm E, Altman DG, Gøtzsche PC, Mulrow CD, Pocock SJ, et al. Strengthening the Reporting of Observational Studies in Epidemiology (STROBE): explanation and elaboration. Epidemiology. 2007 Nov;18(6):805-35. PMID: 18049195. doi: 10.1097/EDE.0b013e3181577511.
