# Supplementary material for: Effect of an Online Continuing Professional Development Course on Physicians’ Intention to Approach a Colleague in Difficulty: Mixed Methods Convergent Study
Source: JMIR Med Educ. 2026 Feb 5;12:e80199. doi: 10.2196/80199 (PMC12921432; doi:10.2196/80199)
Supplement: Multimedia Appendix 3 [file mededu_v12i1e80199_app3.docx]

**Multimedia Appendix 3: Standards for Reporting Qualitative Research (SRQR)**

| No. | Topic | Item | Checklist |
| --- | --- | --- | --- |
| S1 | Title | Concise description of the nature and topic of the study; identifying the study as qualitative or indicating the approach (e.g., ethnography, grounded theory); data collection methods (e.g., interview, focus group) recommended. | Page 1, L1-2 |
| S2 | Abstract | Summary of key elements of the study using the abstract format of the intended publication; typically includes background, purpose, methods, results, and conclusions. | Page 2-3, L36-71 |
| S3 | Problem formulation | Description and significance of the problem/phenomenon studied; review of relevant theory and empirical work; problem statement. | Pages4-5, L103-136 |
| S4 | Purpose or research question | Purpose of the study and specific objectives or questions. | Page 5, L137-140 |
| S5 | Qualitative approach and research paradigm | Qualitative approach (e.g., ethnography, grounded theory, case study); identifying research paradigm (e.g., postpositivist, constructivist/interpretivist); rationale. | Not mentioned |
| S6 | Researcher characteristics and reflexivity | Researchers’ characteristics that may influence the research (e.g., personal attributes, qualifications/experience, relationship with participants); assumptions and presuppositions; potential biases. | Page 11, L326-329 |
| S7 | Context | Setting/site and salient contextual factors; rationale. | Page 6, L167-172    Page 6 L179-208 |
| S8 | Sampling strategy | How and why participants, documents, or events were selected; criteria for deciding when no further sampling was necessary; rationale. | Page 6, L173-175 |
| S9 | Ethical issues pertaining to human subjects | Documentation of approval by ethics review board and participant consent; confidentiality and data security. | Page 12, L371-378 |
| S10 | Data collection methods | Types of data collected; details of data collection procedures (e.g., start/end dates, number of contacts, locations); rationale. | Page 7, L211-220, Pages 8-9 L231-257  Page 9, Figure 2 |
| S11 | Data collection instruments and technologies | Description of instruments (e.g., interview guides) and devices (e.g., audio recorders); changes during the study. | Multimedia Appendices 4 and5  Observation grid: Multimedia Appendix 6 |
| S12 | Units of study | Number and characteristics of participants/documents/events; level of participation. | Page 12-13, L382-398  Page 13, Figure 3 |
| S13 | Data processing | Methods for processing data (e.g., transcription, data entry, management, security, anonymization). | Page 10-11, L318-353 |
| S14 | Data analysis | Process by which inferences, themes, etc., were identified and developed; references to specific paradigms or approaches; rationale. | Page 10. L319-331  Page 11, L46-353 |
| S15 | Techniques to enhance trustworthiness | Techniques to enhance trustworthiness (e.g., member checking, audit trail, triangulation); rationale. | Page 11. L334-338 |
| S16 | Synthesis and interpretation | Main findings (e.g., interpretations, themes); integration with prior research/theory. | Page 20, L510-522  Page 24, L647-654 |
| S17 | Links to empirical data | Evidence (e.g., quotes, field notes, text excerpts, photographs) to substantiate analytic findings. | Pages 19-20, Table 5 |
| S18 | Integration with prior work, implications, transferability, and contribution(s) to the field | Short summary of main findings; explanation of how findings connect to prior work; implications; discussion of scope of applicability; unique contributions. | Pages 25-26, L697-719 |
| S19 | Limitations | Discussion of limitations and their influence on findings. | Not mentioned |
| S20 | Conflicts of interest | Potential sources of influence or perceived influence on study conduct and conclusions. | Page 27, L763 |
| S21 | Funding | Sources of funding and other support; role of funders in data collection, interpretation, and reporting. | Page 27, L759-760 |

Source: O'Brien BC, Harris IB, Beckman TJ, Reed DA, Cook DA. Standards for reporting qualitative research: a synthesis of recommendations. Acad Med. 2014 Sep;89(9):1245-51. PMID: 24979285. doi: 10.1097/acm.0000000000000388.
