# Supplementary material for: Effect of an Online Continuing Professional Development Course on Physicians’ Intention to Approach a Colleague in Difficulty: Mixed Methods Convergent Study
Source: JMIR Med Educ. 2026 Feb 5;12:e80199. doi: 10.2196/80199 (PMC12921432; doi:10.2196/80199)
Supplement: Multimedia Appendix 4 [file mededu_v12i1e80199_app4.docx]

**Multimedia Appendix 4 : Questionnaires et les formulaires de consentement**

Table des matières

[Questionnaire DPC-REACTION administré avant et après le cours de DPC (T1 et T2) 2](#_Toc213579237)

[Formulaire de consentement disponible sur la plateforme MÉDUSE avant le cours de DPC (T1) 3](#_Toc213579238)

[Questionnaire administré avant le cours de DPC (T1) 4](#_Toc213579239)

[Formulaire de consentement disponible sur la plateforme MÉDUSE après le cours de DPC (T2) 6](#_Toc213579240)

[Questionnaire administré après le cours de DPC (T2) 7](#_Toc213579241)

[Formulaire de consentement envoyé 4 mois après le cours de DPC (T3) 9](#_Toc213579242)

[Questionnaire sur le comportement auto-rapporté administré 4 mois après le cours de DPC (T3) 10](#_Toc213579243)

Important note: For the questionnaire translated in English from the original version administered in French, please consult Multimedia Appendix 2.

# Questionnaire DPC-REACTION administré avant et après le cours de DPC (T1 et T2)

Validé en anglais et en français [1, 2], administré en français.

Q1. J’ai l’intention d’approcher un collègue en difficulté.

Fortement en désaccord … Fortement en accord [Échelle de Likert de 1 à 7]

Q2. Au meilleur de ma connaissance, la proportion de mes collègues qui approche un collègue en difficulté est de :

0-20 %

21-40 %

41-60 %

61-80 %

81-100 %

Q3. Je suis confiant que je pourrais approcher un collègue en difficulté si je le voulais.

Fortement en désaccord … Fortement en accord [Échelle de Likert de 1 à 7]

Q4. Il est éthique d’approcher un collègue en difficulté.

Fortement en désaccord … Fortement en accord [Échelle de Likert de 1 à 7]

Q5. Pour moi, approcher un collègue en difficulté serait :

Extrêmement difficile … Extrêmement facile [Échelle de Likert de 1 à 7]

Q6. Pensez maintenant à un collègue de travail que vous respectez comme professionnel. À votre avis, approche-t-il un collègue en difficulté ?

Jamais … Toujours [Échelle de Likert de 1 à 7]

Q7. Je planifie d’approcher un collègue en difficulté.

Fortement en désaccord … Fortement en accord [Échelle de Likert de 1 à 7]

Q8. De façon générale, je pense qu’approcher un collègue en difficulté serait pour moi :

Inutile … Utile [Échelle de Likert de 1 à 7]

Q9. La plupart des personnes importantes pour moi dans ma profession approche un collègue en difficulté.

Fortement en désaccord … Fortement en accord [Échelle de Likert de 1 à 7]

Q10. Il est acceptable d’approcher un collègue en difficulté.

Fortement en désaccord … Fortement en accord [Échelle de Likert de 1 à 7]

Q11. Je suis capable d’approcher un collègue en difficulté.

Fortement en désaccord … Fortement en accord [Échelle de Likert de 1 à 7]

Q12. De façon générale, je pense qu’approcher un collègue en difficulté serait pour moi :

Néfaste … Bénéfique [Échelle de Likert de 1 à 7]

# Formulaire de consentement disponible sur la plateforme MÉDUSE avant le cours de DPC (T1)

Version originale administrée en français.

Cher Docteur

Chère Docteure

La FMSQ souhaite déterminer les retombées potentielles de l’activité de formation « Approcher un collègue en difficulté » sur l’intention d’apporter un changement. Dans le cadre de ce projet de recherche, vous serez invité à compléter trois courts sondages, soit avant et après l’activité de formation, puis 4 mois à la suite de l’activité. Remplir chacun de ces questionnaires ne devrait pas prendre plus de 5 minutes de votre temps.

Votre participation et vos réponses seront confidentielles et anonymes. Votre participation est volontaire et implique votre consentement. Vous pouvez arrêter votre participation à ce projet à n’importe quel stade. Aucune information personnelle ou d’identification ne sera enregistrée dans le cadre de ce projet. Seules les données désidentifiées et regroupées seront rapportées, présentées et publiées.

En cliquant sur « suivant », vous consentez à participer à ce projet.

Pour tout question ou commentaire, veuillez contacter Martin Tremblay PhD, Conseiller principal recherche et innovation pédagogique à la FMSQ, au courriel suivant : [mtremblay@fmsq.org](mailto:mtremblay@fmsq.org).

Merci pour votre précieuse collaboration!

L’équipe de la direction du Développement professionnel continu

# Questionnaire administré avant le cours de DPC (T1)

Version originale administrée en français.

Q1. Quelle est votre profession?

a) Médecin spécialiste

b) Omnipraticien

c) Résident

d) Autre, veuillez préciser : __________________________

Q2. Afin de nous permettre de comparer vos réponses de façon anonyme, veuillez inscrire un code d'identification personnel. Il s'agit de votre année de naissance suivie des initiales de votre mère. Par exemple, si vous êtes né en 1970 et que votre mère s'appelle Anne-Marie Cloutier, alors votre code sera 1970AMC.

Q3. Sexe

a) Homme

b) Femme

c) Autre / non binaire

d) Ne préfère pas divulguer

Q4. Âge (Réponse en nombre) __________________

Q5. Veuillez indiquer de quelle association affiliée à la FMSQ vous faites partie. (*Menu déroulant*)

Association des allergologues et immunologues du Québec

Association des anesthésiologistes du Québec

Association des médecins biochimistes du Québec

Association des cardiologues du Québec

Association des chirurgiens cardio-vasculaires et thoraciques du Québec

Association québécoise de chirurgie

Association de chirurgie vasculaire et endovasculaire du Québec

Association des spécialistes en chirurgie plastique et esthétique du Québec

Association des médecins spécialistes dermatologues du Québec

Association des médecins endocrinologues du Québec

Association des gastro-entérologues du Québec

Association des médecins généticiens du Québec

Association des médecins gériatres du Québec

Association des médecins hématologues et oncologues du Québec

Association des spécialistes en médecine interne du Québec

Association des médecins spécialistes en médecine nucléaire du Québec

Association des médecins microbiologistes infectiologues du Québec

Association des néphrologues du Québec

Association de neurochirurgie du Québec

Association des neurologues du Québec

Association des obstétriciens et gynécologues du Québec

Association des médecins ophtalmologistes du Québec

Association d’orthopédie du Québec

Association d’oto-rhino-laryngologie et de chirurgie cervico-faciale du Québec

Association des pathologistes du Québec

Association des pédiatres du Québec

Association des physiatres du Québec

Association des pneumologues de la province de Québec

Association des médecins psychiatres du Québec

Association des radiologistes du Québec

Association des radio-oncologues du Québec

Association des médecins rhumatologues du Québec

Association des spécialistes en médecine préventive du Québec

Association des spécialistes en médecine d’urgence du Québec

Association des urologues du Québec

Autre

**Q6 à Q17.** Les douze items du questionnaire CPD-REACTION ont été inclus ici (voir la première section détaillant ce questionnaire)

# Formulaire de consentement disponible sur la plateforme MÉDUSE après le cours de DPC (T2)

Version originale administrée en français.

Cher Docteur

Chère Docteure,

La FMSQ souhaite déterminer les retombées potentielles de l’activité de formation « Approcher un collègue en difficulté » sur l’intention d’apporter un changement. Dans le cadre de ce projet de recherche, vous serez invité à compléter trois courts sondages, soit avant et après l’activité de formation, puis 4 mois à la suite de l’activité. Remplir chacun de ces questionnaires ne devrait pas prendre plus de 5 minutes de votre temps.

Votre participation et vos réponses seront confidentielles et anonymes. Votre participation est volontaire et implique votre consentement. Vous pouvez arrêter votre participation à ce projet à n’importe quel stade. Aucune information personnelle ou d’identification ne sera enregistrée dans le cadre de ce projet. Seules les données désidentifiées et regroupées seront rapportées, présentées et publiées. En cliquant sur « suivant », vous consentez à participer à ce projet.

En cliquant sur « suivant », vous consentez à compléter le deuxième sondage de ce projet.

Pour tout question ou commentaire, veuillez contacter Martin Tremblay PhD, Conseiller principal recherche et innovation pédagogique à la FMSQ au courriel suivant : [mtremblay@fmsq.org](mailto:mtremblay@fmsq.org).

Merci pour votre précieuse collaboration!

L’équipe de la direction du Développement professionnel continu

# Questionnaire administré après le cours de DPC (T2)

Version originale administrée en français.

Q1. Veuillez inscrire le code d'identification que vous aviez déterminé au début de cette activité de formation. Il s'agit de votre année de naissance suivie des initiales de votre mère. Par exemple, si vous êtes né en 1970 et que votre mère s'appelle Anne-Marie Cloutier, alors votre code sera 1970AMC.

Q2 à Q13. Les douze questions du DPC RÉACTION étaient incluses ici (voir la première section détaillant ce questionnaire).

Q14. Ce module de formation contenait un ou des messages qui s'appliquent à ma pratique. (*Échelle de Likert à quatre items*)

1 = Tout à fait d’accord

2 = D'accord

3 = En désaccord

4 =Tout à fait en désaccord

8 = Ne s’applique pas

Q15.1. Ce module de formation en ligne vous a-t-il permis d'atteindre vos objectifs d'apprentissage ?

(*Choix binomial : oui ou non*)

Q15.2. Si non, pourquoi? (*Question à réponse ouverte*)

Q16.1. Suite à ce module de formation, prévoyez-vous apporter un changement à votre pratique? (*Choix binomial : oui ou non*)

Q16.2. Pourquoi ? (Que vous ayez l'intention d'apporter ou non un changement à votre pratique) (*Question à réponse ouverte*)

Avis sur le contenu de l'activité

Q17. Il y a absence de conflits d’intérêts dans le contenu du module. (*Choix binomial : oui ou non*)

Q18. Il y a absence de biais commercial dans le contenu du module. (*Choix binomial : oui ou non*)

Q19. Cette activité respecte le Code d'éthique du CQDPCM. (*Choix binomial : oui ou non*)

Q20. Si vous avez noté un manquement au niveau de l'éthique (conflit d'intérêt, biais commercial, non utilisation de noms génériques ou non-respect du code d'éthique, du CQDPCM), pourriez-vous préciser ce que vous avez remarqué ? (*Question à réponse ouverte*)

Q21. Une formation en ligne convient parfaitement au sujet dont il était question. (*Échelle de Likert à quatre items*)

1 = Tout à fait d’accord

2 = D'accord

3 = En désaccord

4 =Tout à fait en désaccord

8 = Ne s’applique pas

Q22. La présentation des cas pratiques m'a permis de mieux intégrer les bonnes pratiques présentées dans ce module. (*Échelle de Likert à quatre items*)

1 = Tout à fait d’accord

2 = D'accord

3 = En désaccord

4 =Tout à fait en désaccord

8 = Ne s’applique pas

Q23. Le choix et la combinaison des méthodes pédagogiques m'ont aidé à mieux comprendre les concepts présentés dans le module. (*Échelle de Likert à quatre items*)

1 = Tout à fait d’accord

2 = D'accord

3 = En désaccord

4 =Tout à fait en désaccord

8 = Ne s’applique pas

Q24. Les consignes d'utilisation et de navigation étaient claires et faciles à suivre. (*Échelle de Likert à quatre items*)

1 = Tout à fait d’accord

2 = D'accord

3 = En désaccord

4 =Tout à fait en désaccord

8 = Ne s’applique pas

Q25. Le contenu était clair et bien structuré. (*Échelle de Likert à quatre items*)

1 = Tout à fait d’accord

2 = D'accord

3 = En désaccord

4 =Tout à fait en désaccord

8 = Ne s’applique pas

Q26. Le contenu du module respectait les objectifs d'apprentissage. (*Échelle de Likert à quatre items*)

1 = Tout à fait d’accord

2 = D'accord

3 = En désaccord

4 =Tout à fait en désaccord

8 = Ne s’applique pas

Q27. Le(s) conférencier(s) maîtrise(nt) bien le sujet.(*Échelle de Likert à quatre items*)

1 = Tout à fait d’accord

2 = D'accord

3 = En désaccord

4 =Tout à fait en désaccord

8 = Ne s’applique pas

Q28.1. Recommanderiez-vous ce module à vos collègues ? (*Choix binomial : oui ou non*)

Q28.2. Si non, pourquoi? (*Question à réponse ouverte*)

Q29. Selon vous, quels sont les points forts de cette activité ? (*Question à réponse ouverte*)

Q30. Selon vous, quels sont les aspects de cette activité qui devraient être améliorés ? (*Question à réponse ouverte*)

Q31. Quel(s) sujet(s) auriez-vous besoin d’aborder lors de vos prochaines activités de DPC ? (*Question à réponse ouverte*)

# Formulaire de consentement envoyé 4 mois après le cours de DPC (T3)

Version originale envoyée en français.

Titre du courriel : Formation « Approcher un collègue en difficulté » - suivi à 4 mois

Contenu du courriel

Cher Docteur

Chère Docteure

La FMSQ souhaite déterminer les retombées potentielles de l’activité de formation « Approcher un collègue en difficulté » sur l’intention d’apporter un changement. Dans le cadre de ce projet de recherche, vous serez invité à compléter trois courts sondages, soit avant et après l’activité de formation, puis 4 mois à la suite de l’activité.

Veuillez prendre quelques minutes afin de compléter le dernier sondage en cliquant sur le lien ci-dessous. En cliquant sur « suivant », vous consentez à participer à ce projet. Répondre à ce sondage de 4 questions ne devrait pas prendre plus de 3 minutes de votre temps.

Pour tout question ou commentaire, veuillez contacter Martin Tremblay PhD, Conseiller principal recherche et innovation pédagogique à la FMSQ au courriel suivant : [mtremblay@fmsq.org](mailto:mtremblay@fmsq.org).

Merci pour votre précieuse collaboration!

L’équipe de la direction du Développement professionnel continu

# Questionnaire sur le comportement auto-rapporté administré 4 mois après le cours de DPC (T3)

Version originale administrée en français.

Q1. Veuillez inscrire le code d'identification que vous aviez déterminé au début de cette activité de formation. Il s'agit de votre année de naissance suivie des initiales de votre mère. Par exemple, si vous êtes né en 1970 et que votre mère s'appelle Anne-Marie Cloutier, alors votre code sera 1970AMC.

Q2. Avez-vous approché un collègue en difficulté au cours des 4 derniers mois en suivant les apprentissages que vous avez acquis lors de la formation du même nom sur MÉDUSE ?

a) Oui

b) Non, veuillez expliquer pourquoi : __________ (*Question à réponse ouverte*)

Q3. Selon vous, votre cours de formation a-t-il eu des retombées sur la sécurité ou la santé de vos patients ou ceux de la personne approchée? (Réponse à choix multiples)

a) Oui, veuillez expliquer en donnant un exemple précis : __________ (*Question à réponse ouverte*)

b) Non, veuillez expliquer pourquoi : __________ (*Question à réponse ouverte*)

c) Sans objet

Q4. Accepteriez-vous de participer à une courte entrevue téléphonique avec les investigateurs de ce projet de recherche pour nous faire part de votre expérience?

a) Oui, veuillez écrire votre nom et courriel : __________

b) Non

Reference

1. Légaré F, Borduas F, Freitas A, Jacques A, Godin G, Luconi F, Grimshaw J. Development of a simple 12-item theory-based instrument to assess the impact of continuing professional development on clinical behavioral intentions. PLoS One. 2014;9(3):e91013. PMID: 24643173. doi: 10.1371/journal.pone.0091013.

2. Légaré F, Borduas F, Freitas A, Turcotte S. User Manuel-The Continuing Professional Development (CPD) Reaction Questionnaire. 2015.
