# Supplementary material for: Effect of an Online Continuing Professional Development Course on Physicians’ Intention to Approach a Colleague in Difficulty: Mixed Methods Convergent Study
Source: JMIR Med Educ. 2026 Feb 5;12:e80199. doi: 10.2196/80199 (PMC12921432; doi:10.2196/80199)
Supplement: Multimedia Appendix 5 [file mededu_v12i1e80199_app5.docx]

**Multimedia Appendix 5: Complete Questionnaires and Consent Forms**

Table of content

[CPD-REACTION Questionnaire Administered Before and After the CPD Course (T1 and T2) 2](#_Toc214129096)

[Consent Form Delivered Before the CPD Course (T1) via the MEDUSE Platform 3](#_Toc214129097)

[Questionnaire Administered Before the CPD Course (T1) 4](#_Toc214129098)

[Consent Form Delivered After the CPD Course (T2) via the MEDUSE Platform 6](#_Toc214129099)

[Questionnaire Administered After the CPD Course (T2) 7](#_Toc214129100)

[Consent Form Sent 4 Months After the CPD Course (T3) 9](#_Toc214129101)

[Questionnaire on Self-reported Behavior Sent 4 Months After the CPD Course (T3) 10](#_Toc214129102)

Important note: This is a translated version. For the original version administered in French, please consult Multimedia Appendix 3.

# CPD-REACTION Questionnaire Administered Before and After the CPD Course (T1 and T2)

Validated in English and in French [1, 2], administered in French.

**Q1**. I intend to approach a colleague in difficulty.

Strongly disagree … Strongly agree [Likert scale 1 to 7]

**Q2**. To the best of my knowledge, the percentage of my colleagues who approach a colleague in difficulty is:

0-20 %

21-40 %

41-60 %

61-80 %

81-100 %

**Q3**. I am confident that I could approach a colleague in difficulty if I wanted to.

Strongly disagree … Strongly agree [Likert scale 1 to 7]

**Q4**. Approaching a colleague in difficulty is the ethical thing to do.

Strongly disagree … Strongly agree [Likert scale 1 to 7]

**Q5**. For me, approaching a colleague in difficulty would be:

Extremely difficult … Extremely easy [Likert scale 1 to 7]

**Q6**. Now think about a co-worker whom you respect as a professional. In your opinion, does he/she approach a colleague in difficulty?

Never … Always [Likert scale 1 to 7]

**Q7.** I plan to approach a colleague in difficulty.

Strongly disagree … Strongly agree [Likert scale 1 to 7]

**Q8.** Overall, I think that for me approaching a colleague in difficulty would be:

Useless … Useful [Likert scale 1 to 7]

**Q9.** Most people who are important to me in my profession approach a colleague in difficulty.

Strongly disagree … Strongly agree [Likert scale 1 to 7]

**Q10.** It is acceptable to approach a colleague in difficulty.

Strongly disagree … Strongly agree [Likert scale 1 to 7]

**Q11.** I have the ability to approach a colleague in difficulty.

Strongly disagree … Strongly agree [Likert scale 1 to 7]

**Q12.** Overall, I think that for me approaching a colleague in difficulty would be:

Harmful … Beneficial [Likert scale 1 to 7]

# Consent Form Delivered Before the CPD Course (T1) via the MEDUSE Platform

Translated in English from the original version administered in French.

Dear Doctor,

The FMSQ wishes to determine the potential impact of the training activity “Approaching a Colleague in Difficulty” on the intention to adopt this new behavior. As part of this research project, you will be invited to complete three short questionnaires: one before the training activity, one after, and one four months following the activity. Completing each of these questionnaires should take no more than 5 minutes of your time.

Your participation and responses will be confidential and anonymous. Participation is voluntary and implies your consent. You may withdraw from the project at any time. No personal or identifying information will be recorded as part of this project. Only de-identified and aggregated data will be reported, presented, and published.

By clicking “Next,” you consent to participate to this project.

For any questions or comments, please contact Martin Tremblay, PhD, Senior Advisor for Research and Educational Innovation at the FMSQ, at the following email: mtremblay@fmsq.org.

Thank you for your valuable collaboration!

The Continuing Professional Development Team

# Questionnaire Administered Before the CPD Course (T1)

Translated in English from the original version administered in French.

Q1. What is your profession?
a) Medical specialist
b) General practitioner
c) Resident
d) Other, please specify: __________________________

Q2. To allow us to compare your responses anonymously, please enter a personal identification code.
This code consists of your year of birth followed by your mother's initials.
For example, if you were born in 1970 and your mother’s name is Anne-Marie Cloutier, your code would be 1970AMC.

Q3. Gender
a) Male
b) Female
c) Other / Non-binary
d) Prefer not to disclose

Q4. Age (Numeric response) __________________

Q5. Please indicate which FMSQ affiliated association you belong to. (Dropdown menu)

Quebec Association of Allergists and Immunologists

Quebec Association of Anesthesiologists

Quebec Association of Medical Biochemists

Quebec Association of Cardiologists

Quebec Association of Cardiovascular and Thoracic Surgeons

Quebec Surgical Association

Quebec Association of Vascular and Endovascular Surgery

Quebec Association of Plastic and Aesthetic Surgery Specialists

Quebec Association of Dermatology Specialists

Quebec Association of Endocrinologists

Quebec Association of Gastroenterologists

Quebec Association of Medical Geneticists

Quebec Association of Geriatricians

Quebec Association of Hematologists and Oncologists

Quebec Association of Internal Medicine Specialists

Quebec Association of Nuclear Medicine Specialists

Quebec Association of Medical Microbiologists and Infectiologists

Quebec Association of Nephrologists

Quebec Association of Neurosurgeons

Quebec Association of Neurologists

Quebec Association of Obstetricians and Gynecologists

Quebec Association of Ophthalmologists

Quebec Orthopedic Association

Quebec Association of Otolaryngologists and Head and Neck Surgeons

Quebec Association of Pathologists

Quebec Association of Pediatricians

Quebec Association of Physiatrists

Quebec Association of Pulmonologists

Quebec Association of Psychiatrists

Quebec Association of Radiologists

Quebec Association of Radiation Oncologists

Quebec Association of Rheumatologists

Quebec Association of Preventive Medicine Specialists

Quebec Association of Emergency Medicine Specialists

Quebec Association of Urologists

Other

**Q6 to Q17.** The twelve items of the CPD-REACTION questionnaire were included here (see first section detailing this questionnaire).

# Consent Form Delivered After the CPD Course (T2) via the MEDUSE Platform

Translated in English from the original version administered in French.

Dear Doctor,

The FMSQ wishes to determine the potential impact of the training activity “Approaching a Colleague in Difficulty” on the intention to adopt this new behavior. As part of this research project, you will be invited to complete three short questionnaires: one before the training activity, one after, and one four months following the activity. Completing each of these questionnaires should take no more than 5 minutes of your time.

Your participation and responses will be confidential and anonymous. Participation is voluntary and implies your consent. You may withdraw from the project at any time. No personal or identifying information will be recorded as part of this project. Only de-identified and aggregated data will be reported, presented, and published. By clicking “Next,” you consent to participate to this project.

By clicking “Next,” you also consent to complete the second questionnaire of this project.

For any questions or comments, please contact Martin Tremblay, PhD, Senior Advisor for Research and Educational Innovation at the FMSQ, at the following email: mtremblay@fmsq.org.

Thank you for your valuable collaboration!

The Continuing Professional Development Team

# Questionnaire Administered After the CPD Course (T2)

Translated in English from the original version administered in French.

**Q1.** Please enter the identification code you determined at the beginning of this training activity. It consists of your year of birth followed by your mother’s initials. For example, if you were born in 1970 and your mother’s name is Anne-Marie Cloutier, then your code will be **1970AMC**.

**Q2 to Q13.** The twelve items of the CPD-REACTION questionnaire were included here (see first section detailing this questionnaire).

**Q14.** This training module contained one or more messages that apply to my practice. *(Four-point Likert scale)*
1 = Strongly agree
2 = Agree
3 = Disagree
4 = Strongly disagree
8 = Not applicable

**Q15.1.** Did this online training module allow you to achieve your learning objectives?
*(Binary choice: Yes or No)*

**Q15.2.** If no, why? *(Open-ended question)*

**Q16.1.** After this training module, do you plan to make a change to your practice?
*(Binary choice: Yes or No)*

**Q16.2.** Why? *(Whether or not you intend to make a change to your practice)* *(Open-ended question)*

**Q17.** There is no conflict of interest in the module content. *(Binary choice: Yes or No)*

**Q18.** There is no commercial bias in the module content. *(Binary choice: Yes or No)*

**Q19.** This activity complies with the CQDPCM Code of Ethics. *(Binary choice: Yes or No)*

**Q20.** If you noted an ethical issue (conflict of interest, commercial bias, failure to use generic names, or non-compliance with the CQDPCM Code of Ethics), could you specify what you observed? *(Open-ended question)*

**Q21.** An online training format is perfectly suited to the topic addressed. *(Four-point Likert scale)*
1 = Strongly agree
2 = Agree
3 = Disagree
4 = Strongly disagree
8 = Not applicable

**Q22.** The presentation of practical cases helped me better integrate the best practices presented in this module. *(Four-point Likert scale)*
1 = Strongly agree
2 = Agree
3 = Disagree
4 = Strongly disagree
8 = Not applicable

**Q23.** The choice and combination of teaching methods helped me better understand the concepts presented in the module. *(Four-point Likert scale)*
1 = Strongly agree
2 = Agree
3 = Disagree
4 = Strongly disagree
8 = Not applicable

**Q24.** The usage and navigation instructions were clear and easy to follow. *(Four-point Likert scale)*
1 = Strongly agree
2 = Agree
3 = Disagree
4 = Strongly disagree
8 = Not applicable

**Q25.** The content was clear and well-structured. *(Four-point Likert scale)*
1 = Strongly agree
2 = Agree
3 = Disagree
4 = Strongly disagree
8 = Not applicable

**Q26.** The module content met the learning objectives. *(Four-point Likert scale)*
1 = Strongly agree
2 = Agree
3 = Disagree
4 = Strongly disagree
8 = Not applicable

**Q27.** The speaker(s) demonstrated mastery of the subject. *(Four-point Likert scale)*
1 = Strongly agree
2 = Agree
3 = Disagree
4 = Strongly disagree
8 = Not applicable

**Q28.1.** Would you recommend this module to your colleagues? *(Binary choice: Yes or No)*

**Q28.2.** If no, why? *(Open-ended question)*

**Q29.** In your opinion, what are the strengths of this activity? *(Open-ended question)*

**Q30.** In your opinion, what aspects of this activity should be improved? *(Open-ended question)*

**Q31.** What topic(s) would you need to address in your next CPD activities? *(Open-ended question)*

# Consent Form Sent 4 Months After the CPD Course (T3)

Translated in English from the original version administered in French.

Email Subject: *Training “Approaching a Colleague in Difficulty” – 4-Month Follow-Up*

Email Content:
Dear Doctor,

The FMSQ wishes to determine the potential impact of the training activity *“Approaching a Colleague in Difficulty”* on the intention to adopt this new behavior. As part of this research project, you will be invited to complete three short questionnaires: one before the training activity, one after, and one four months following the activity.

Please take a few minutes to complete the final questionnaire by clicking on the link below. By clicking “Next,” you consent to participate in this project. Answering this 4-question questionnaire should take no more than 3 minutes of your time.

For any questions or comments, please contact Martin Tremblay, PhD, Senior Advisor for Research and Educational Innovation at the FMSQ, at the following email: mtremblay@fmsq.org.

Thank you for your valuable collaboration!

The Continuing Professional Development Team

# Questionnaire on Self-reported Behavior Sent 4 Months After the CPD Course (T3)

Translated in English from the original version administered in French.

**Q1.** Please enter the identification code you determined at the beginning of this training activity. It consists of your year of birth followed by your mother’s initials. For example, if you were born in 1970 and your mother’s name is Anne-Marie Cloutier, then your code will be **1970AMC**.

**Q2.** In the past 4 months, have you approached a colleague in difficulty by applying what you learned during the training of the same name on MÉDUSE?
a) Yes
b) No, please explain why: __________ *(Open-ended question)*

**Q3.** In your opinion, did your training course have an impact on the safety or health of your patients or those of the colleague you approached?
a) Yes, please explain by giving a specific example: __________
b) No, please explain why: __________ *(Open-ended question)*
c) Not applicable

**Q4.** Would you agree to participate in a short telephone interview with the investigators of this research project to share your experience?
a) Yes, please provide your name and email: __________
b) No

References

1. Légaré F, Borduas F, Freitas A, Jacques A, Godin G, Luconi F, Grimshaw J. Development of a simple 12-item theory-based instrument to assess the impact of continuing professional development on clinical behavioral intentions. PLoS One. 2014;9(3):e91013. PMID: 24643173. doi: 10.1371/journal.pone.0091013.

2. Légaré F, Borduas F, Freitas A, Turcotte S. User Manuel-The Continuing Professional Development (CPD) Reaction Questionnaire. 2015.
