# Supplementary material for: Effect of an Online Continuing Professional Development Course on Physicians’ Intention to Approach a Colleague in Difficulty: Mixed Methods Convergent Study
Source: JMIR Med Educ. 2026 Feb 5;12:e80199. doi: 10.2196/80199 (PMC12921432; doi:10.2196/80199)
Supplement: Multimedia Appendix 6 [file mededu_v12i1e80199_app6.docx]

**Multimedia Appendix 6: Grid for evaluating the CPD course "Approaching a Colleague in Difficulty" offered by the Fédération des médecins spécialistes du Québec (FMSQ) according to Michie’s Behavior Change Techniques**

Evaluator's Name:

Evaluation Date:

| **Behavior Change Technique** | **Definition** | **Evaluation (present/absent)** |
| --- | --- | --- |
| Goal/target specified: behaviour or outcome | Clearly indicate the targeted behavior [1]. |  |
| Monitoring | Recording the individual's behavior and performance [1]. |  |
| Self-monitoring | Keeping a record of one's behavior (time, place of behavior, feelings) [1, 2]. |  |
| Contract | Agreed performance of the target behavior with at least one other person, written and signed [1]. |  |
| Rewards; incentives (inc. self-evaluation) | Valued consequence conditional on adopting the behavior, including social approval conditional on adopting the behavior [1]. |  |
| Graded task, starting with easy tasks | Start with easy tasks, gradually making them more difficult until the target behavior is achieved [1]. |  |
| Increasing skills: problem-solving, decision-making, goal-setting | Problem-solving, decision-making, goal setting [1]. |  |
| Stress management | Strategies adopted to reduce stress factors or the impact of stress factors [1]. |  |
| Coping skills | Behaviors adopted to avoid stressors or stress triggers [1]. |  |
| Rehearsal of relevant skills | Performing the behavior (repeatedly) [1]. |  |
| Role-play | Performing the behavior in a simulated situation [1]. |  |
| Planning, implementation | Identifying the components of the behavior and establishing a plan to execute each of them or considering when and/or where a behavior will be performed, i.e., scheduling behaviors [1]. |  |
| Prompts, triggers, cues | Stimulus that provokes a behavior (including phone calls or postal reminders designed to prompt behavior) [1]. |  |
| Environmental changes (e.g. objects to facilitate behaviour) | Modifying the environment to facilitate the target behavior (other than through rewards and punishments) [1]. |  |
| Social processes of encouragement, pressure, support | Social pressure to adopt the behavior [1]. |  |
| Persuasive communication | A credible source presents arguments in favor of the behavior. Note, there must be a presentation of arguments; general pro-behavior communication does not count [1]. |  |
| Information regarding behaviour, outcome | Providing information on the antecedents or consequences of the behavior, or on the connections between them, or on behavior change techniques [1]. |  |
| Personalised message | Adapting techniques or messages from others to the individual's context, including the person's stage of change [1]. |  |
| Modeling | Observing the behavior of others which act as a demonstration [1]. |  |
| Homework | Defining tasks to do at home [1]. |  |
| Personal experiments, data collection (other than self-monitoring of behaviour) | Asking people to test hypotheses about the behavior, its causes, and its consequences, by collecting and interpreting data. This includes personal experiences [1]. |  |
| Experiential: tasks to gain experiences to change motivation | Tasks aimed at acquiring experiences to change motivation [1]. |  |
| Feedback | Providing feedback on monitored behavior (including self-monitored behavior) [1]. |  |
| Self talk | Planned affirmations made to oneself (aloud or silently) to implement behavior change techniques [1]. |  |
| Use of imagery | Use of planned images (visual, motor, sensory) to implement behavior change techniques (including mental rehearsal) [1]. |  |
| Perform behaviour in different settings | Performing the behavior in different contexts [1]. |  |
| Shaping of behaviour | Developing the behavior by initially reinforcing the behavior closest to the required behavior and systematically modifying the behavior needed to obtain conditioned reinforcement [1]. |  |
| Motivational interviewing | Eliciting self-motivational statements and evaluating one's own behavior to reduce resistance to change [1]. |  |
| Relapse prevention | Identifying situations that increase the likelihood that the behavior will not be performed and applying coping strategies to these situations [1]. |  |
| Cognitive restructuring | Changing cognitions about the causes and consequences of the behavior [1]. |  |
| Relaxation | Systematic instruction in physical and cognitive strategies to reduce sympathetic activation, and increase muscle relaxation and the feeling of calm [1]. |  |
| Disinhibition | Exposure to threatening experiences [1]. |  |
| Problem-solving | "The process by which individuals attempt to overcome difficulties, achieve goals that move them from an initial situation to a desired goal, or arrive at conclusions through the use of higher mental functions such as reasoning and creative thinking. [3]" |  |
| Time management | Planning actions applied to the perceived problem of lack of time [1]. |  |
| Identify/prepare for difficult situation/problems | Identifying and planning ways to overcome obstacles (note that this must include identifying a specific obstacle)[1]. |  |

**Evaluator's Notes**

| Behavior Change Technique | Notes on evaluation |
| --- | --- |
|  |  |
|  |  |
|  |  |
|  |  |
|  |  |
|  |  |
|  |  |
|  |  |
|  |  |
|  |  |
|  |  |
|  |  |
|  |  |

References

1. Michie S, Johnston M, Francis J, Hardeman W, Eccles M. From Theory to Intervention: Mapping Theoretically Derived Behavioural Determinants to Behaviour Change Techniques. Applied Psychology. 2008;57(4):660-80. doi: 10.1111/j.1464-0597.2008.00341.x.

2. Sutton S. Interventions très brèves sur le changement de comportement dans des environnements de soins de santé. Practical Health Psychology; 2017; Available from: <https://practicalhealthpsychology.com/fr/2017/11/very-brief-interventions/>.

3. Reed SK. The Oxford handbook of cognitive science. Oxford University Press. 2017:231-247.
