# Supplementary material for: Effect of an Online Continuing Professional Development Course on Physicians’ Intention to Approach a Colleague in Difficulty: Mixed Methods Convergent Study
Source: JMIR Med Educ. 2026 Feb 5;12:e80199. doi: 10.2196/80199 (PMC12921432; doi:10.2196/80199)
Supplement: Multimedia Appendix 7 [file mededu_v12i1e80199_app7.docx]

**Multimedia Appendix 7: Number and percentage of participants with missing data for each variable**

*Table S1*. Participants with missing data for each variable questionnaire at T1, before the CPD course (n= 665)

| Variable | Number of missing entries | % of missing data |
| --- | --- | --- |
| Age | 0 | 0 |
| Gender | 0 | 0 |
| Domain of practice | 0 | 0 |
| Item 1 | 1 | 0.15 |
| Item 2 | 1 | 0.15 |
| Item 3 | 2 | 0.3 |
| Item 4 | 0 | 0 |
| Item 5 | 3 | 0.45 |
| Item 6 | 1 | 0.15 |
| Item 7 | 1 | 0.15 |
| Item 8 | 6 | 0.9 |
| Item 9 | 1 | 0.15 |
| Item 10 | 3 | 0.45 |
| Item 11 | 4 | 0.6 |
| Item 12 | 1 | 0.15 |

*Table S2.* Participants with missing data for each variable questionnaire at T2, after the CPD course (n=563)

| Variable | Number of missing entries | % of missing data |
| --- | --- | --- |
| Age | 97 ^(a)^ | 17.2 |
| Gender | 97 ^(a)^ | 17.2 |
| Domain of practice | 97 ^(a)^ | 17.2 |
| Item 1 | 1 | 0.18 |
| Item 2 | 0 | 0 |
| Item 3 | 1 | 0.18 |
| Item 4 | 2 | 0.36 |
| Item 5 | 0 | 0 |
| Item 6 | 0 | 0 |
| Item 7 | 6 | 1.07 |
| Item 8 | 0 | 0 |
| Item 9 | 1 | 0.18 |
| Item 10 | 3 | 0.53 |
| Item 11 | 4 | 0.71 |
| Item 12 | 0 | 0 |

(a) We do not have the socio-demographic characteristics of the 97 physicians who responded only to the T2 questionnaire without responding to the T1 questionnaire. This structurally missing data occurred because socio-demographic characteristics were not part of the questionnaire at T2.
