# Supplementary material for: Effect of an Online Continuing Professional Development Course on Physicians’ Intention to Approach a Colleague in Difficulty: Mixed Methods Convergent Study
Source: JMIR Med Educ. 2026 Feb 5;12:e80199. doi: 10.2196/80199 (PMC12921432; doi:10.2196/80199)
Supplement: Multimedia Appendix 8 [file mededu_v12i1e80199_app8.docx]

**Multimedia Appendix 8: Assessing Robustness in Bivariate Comparisons**

*Table S1.* Estimation of the effect of the CPD course on the intention to approach a colleague in difficulty

| Variables ^(a)^ | n | β | CI 95% | *P*-value |
| --- | --- | --- | --- | --- |
| CPD-course | 456 | 1.06 | 0.93 ; 1.20 | <.001 |
| Beliefs about capabilities | 457 | 0.37 | 0.25 ; 0.50 | <.001 |
| Social influence | 457 | 0.22 | 0.08 ; 0.36 | .002 |
| Moral norm | 457 | -0.11 | -0.24 ; 0.02 | .10 |
| Beliefs about consequences | 457 | 0.15 | 0.01 ; 0.29 | .03 |
| Age (reference 35 to 54 years of age) | 457 | 0 |  |  |
| < 35 years of age | 457 | -0.11 | -0.45 ; 0.23 | .54 |
| > 54 years of age | 457 | -0.18 | -0.43 ; 0.08 | .18 |
| Domaine (reference surgical) | 457 | 0 |  |  |
| Laboratory | 457 | 0.17 | -0.16 ; 0.50 | .31 |
| Medical | 457 | 0.13 | -0.13 ; 0.39 | .33 |
| Family medicine | 457 | 0.56 | -0.35 ; 1.47 | .23 |
| Other | 457 | -0.01 | -0.83 ; 0.82 | .99 |
| Gender (reference women) | 457 | 0 |  |  |
| Men | 457 | -0.36 | -0.59 ; -0.13 | .002 |
| Other/non-binary/prefer not to disclose | 457 | -0.28 | -1.29 ; 0.73 | .59 |

1. We included determinants of intention measured before the course.

*Table S2.* Sensitivity analysis of comparison of behavioral intention before and after the CPD course Wilcoxon test

| CPD-REACTION constructs^a^ |  | Before CPD^b^ course | After CPD^b^ course | Before versus after CPD^b^ course | | | |
| --- | --- | --- | --- | --- | --- | --- | --- |
|  | n | Mean (SD^c^) | Mean (SD^c^) | Mean Difference (CSD^d^) | CI 95% | *P*-value^e^ | *P*-value^f^ |
| Intention | 455 | 3.88 (1.72) | 4.92 (1.40) | 1.04 (1.45) | 0.91; 1.17 | <.001 | <.0001 |
| Beliefs about capabilities | 459 | 4.65 (1.17) | 5.49 (0.74) | 0.85 (1.11) | 0.74; 0.95 | <.001 | <.0001 |
| Moral norm | 462 | 6.09 (1.04) | 5.79 (0.77) | -0.30 (1.06) | - 0.40; -0.21 | <.001 | <.0001 |
| Social influences | 464 | 3.36 (0.99) | 3.81 (0.99) | 0.45 (0.74) | 0.38; 0.52 | <.001 | <.0001 |
| Beliefs about consequences | 465 | 5.53 (1.04) | 5.67 (0.65) | 0.14 (0.90) | 0.06; 0.22 | <.001 | 0.0006 |

^a^ Response scale of CPD-REACTION questionnaire ranging from 1 to 7

^b^ CPD: Continuing professional development

^c^ SD: Standard deviation

^d^ CSD: Combined Standard deviation

^e^ Paired t-test

^f^ Wilcoxon signed-rank test

*P*-values did not differ significantly whether the paired *t*-test or the Wilcoxon signed-rank test was used to compute these values.

*Table S3.* Mean intention and determinants of intention according to participant response pattern

|  | Participants who completed questionnaire only before the CPD course | Participants who completed questionnaires before and after the CPD course | Participants who completed questionnaire only after the CPD course |
| --- | --- | --- | --- |
| CPD-REACTION constructs before course* | n=199 | n=466 | n=97 |
| Intention | 3.9 (1.6) | 3. 9 (1.7) | NA |
| Beliefs about capabilities | 4.7 (1.2) | 4.6 (1.2) | NA |
| Moral norm | 5.9 (1.3) | 6.1 (1.0) | NA |
| Social influences | 3.4 (1.1) | 3.4 (1.0) | NA |
| Beliefs about consequences | 5.4 (1.2) | 5.5 (1.0) | NA |
| CPD-REACTION constructs after course* | n=199 | n=466 | n=97 |
| Intention | NA | 4.9 (1.4) | 5.0 (1.3) |
| Beliefs about capabilities | NA | 5.5 (0.7) | 5.2 (1.0) |
| Moral norm | NA | 5.8 (0.8) | 5.7 (1.0) |
| Social influences | NA | 3.8 (1.0) | 3.9 (1.0) |
| Beliefs about consequences | NA | 5.7 (0.7) | 5.5 (0.8) |

As shown in *Table S3* above, mean intention and its determinants are similar among the groups with different response patterns.
