# Supplementary material for: Effect of an Online Continuing Professional Development Course on Physicians’ Intention to Approach a Colleague in Difficulty: Mixed Methods Convergent Study
Source: JMIR Med Educ. 2026 Feb 5;12:e80199. doi: 10.2196/80199 (PMC12921432; doi:10.2196/80199)
Supplement: Multimedia Appendix 9 [file mededu_v12i1e80199_app9.docx]

**Multimedia Appendix 9: Analysis of mean intention difference by age, gender and by domain of medical specialty**

Table S1. Stratification of mean intention difference by age, gender and by domain of medical specialty

| Socio-demographic characteristics of physicians | n | Mean intention before CPD course (SD) | Mean intention after CPD course (SD) | Mean intention difference | IC 95% | *P*-value^a^ | *P*-value^b^ |
| --- | --- | --- | --- | --- | --- | --- | --- |
| All physicians | 455 | 3.88 (1.72) | 4.92 (1.40) | 1.04 | 0.91; 1.17 | <.001 | <.001 |
| Age |  |  |  |  |  |  |  |
| < 35 years of age | 64 | 3.87 (1.83) | 4.87 (1.45) | 1.00 | 0.66; 1.34 | <.001 | <.001 |
| 35 to 54 years of age | 239 | 3.93 (1.73) | 5.01 (1.30) | 1.10 | 0.92; 1.27 | <.001 | <.001 |
| > 54 years of age | 152 | 3.83 (1.67) | 4.79 (1.52) | 0.98 | 0.72; 1.23 | <.001 | <.001 |
| Gender |  |  |  |  |  |  |  |
| Women | 234 | 4.06 (1.74) | 5.10 (1.31) | 1.04 | 0.86; 1.21 | <.001 | <.001 |
| Men | 218 | 3.67 (1.69) | 4.73 (1.48) | 1.06 | 0.85; 1.27 | <.001 | <.001 |
| Other/non-binary/prefer not to disclose | 3 | 4.0 (0.0) | 4.33 (0.58) | 0.33 | 1.10; 1.77 | >.99 | .51 |
| Medical specialty domain |  |  |  |  |  |  |  |
| Surgical specialty | 204 | 3.72 (1.83) | 4.79 (1.49) | 1.05 | 0.85; 1.26 | <.001 | <.001 |
| Laboratory specialty | 69 | 3.86 (1.60) | 4.85 (1.47) | 1 | 0.64; 1.37 | <.001 | <.001 |
| Medical specialty | 164 | 4.00 (1.59) | 5.10 (1.24) | 1.11 | 0.90; 1.33 | <.001 | <.001 |
| General practitioner | 9 | 5.44 (1.89) | 5.39 (1.36) | -0.06 | -0.79; 0.67 | .81 | .56 |
| Non-specified specialty | 9 | 4.11 (1.76) | 4.78 (1.30) | 0.66 | 0.00; 1.33 | .11 | .44 |

^a^ Paired *t*-test

^b^ Wilcoxon test for signed ran

Table S2. Effect of age, gender and domain on intention difference

| Socio-demographic characteristics | DF | F value | *P*-value of F value | |
| --- | --- | --- | --- | --- |
| R^2^ adj=0.017 | | | | |
| Medical specialty domain categories | 4 | 1.57 | | .18 |
| Age categories | 2 | 0.27 | | .76 |
| Gender categories | 2 | 0.41 | | .66 |
