# Supplementary material for: Effect of an Online Continuing Professional Development Course on Physicians’ Intention to Approach a Colleague in Difficulty: Mixed Methods Convergent Study
Source: JMIR Med Educ. 2026 Feb 5;12:e80199. doi: 10.2196/80199 (PMC12921432; doi:10.2196/80199)
Supplement: Multimedia Appendix 10 [file mededu_v12i1e80199_app10.docx]

**Multimedia Appendix 10: Sensitivity analysis of predictive factors of physicians’ intention to approach a colleague**

*Table S1.* Sensitivity analysis - Full model of predictive factors of physicians’ intention to approach a colleague experiencing difficulties among all participants who completed the CPD-REACTION questionnaire after the CPD course

| Determinants of intention | n | β | CI 95% | *P*-value |
| --- | --- | --- | --- | --- |
| **R^2^=0.30** | | | | |
| Beliefs about capabilities | 552 | 0.42 | 0.20 ; 0.64 | <.001 |
| Social influence | 552 | 0.27 | 0.16 ; 0.38 | <.001 |
| Moral norm | 552 | 0.27 | 0.08 ; 0.45 | .004 |
| Beliefs about consequences | 552 | 0.20 | 0.01 ; 0.39 | .04 |
| Age (reference 35 to 54 years of age) | 552 |  |  | .35^a^ |
| < 35 years of age |  | -0.22 | -0.55 ; 0.10 | .18 |
| > 54 years of age |  | -0.11 | -0.35 ; 0.13 | .38 |
| Gender (reference women) | 552 |  |  | .53^a^ |
| Men |  | -0.11 | -0.34 ; 0.11 | .34 |
| Other/non-binary/prefer not to disclose |  | -0.45 | -1.79 ; 0.90 | .52 |
| Domaine (reference surgical) | 552 |  |  | .20^a^ |
| Laboratory |  | 0.23 | -0.09 ; 0.56 | .16 |
| Medical |  | 0.28 | 0.04 ; 0.53 | .02 |
| Family medicine |  | 0.39 | -0.40 ; 1.19 | .33 |
| Other |  | 0.30 | -0.49 ; 1.09 | .46 |

^a^ Global p-value

*Table S2.* Sensitivity analysis - Full model of predictive factors of physicians’ intention to approach a colleague experiencing difficulties among all participants who completed the CPD-REACTION questionnaire after the CPD course

| Determinants of intention | n | β | CI 95% | *P*-value |
| --- | --- | --- | --- | --- |
| **R^2^=0.27** | | | | |
| Belief about capabilities | 552 | 0.49 | 0.30; 0.68 | <.001 |
| Social influence | 552 | 0.30 | 0.19; 0.41 | <.001 |
| Moral norm | 552 | 0.28 | 0.10; 0.46 | .002 |

*Table S3.* Pearson correlation coefficient between determinants of intention for multivariate analysis

| Variables being correlated | Pearson correlation coefficient |
| --- | --- |
| Belief about capabilities with moral norm | 0.72 |
| Belief about capabilities with belief about consequences | 0.62 |
| Belief about consequences with moral norm | 0.40 |
| Belief about capabilities with social influence | 0.36 |
| Belief about consequences with social influence | 0.29 |
| Moral norm with social influence | 0.20 |
