# Supplementary material for: Effect of an Online Continuing Professional Development Course on Physicians’ Intention to Approach a Colleague in Difficulty: Mixed Methods Convergent Study
Source: JMIR Med Educ. 2026 Feb 5;12:e80199. doi: 10.2196/80199 (PMC12921432; doi:10.2196/80199)
Supplement: Multimedia Appendix 11 [file mededu_v12i1e80199_app11.docx]

**Multimedia Appendix 11: Supplementary qualitative results**

*Table S1.* Description of the behavioral intention of participants

| Intention for new behavior | Representative excerpts of quotations. | n= 153 ^(a)^ |
| --- | --- | --- |
| More attention or observation of colleagues' signs of difficulty | "More attention to colleagues." "I will be more attentive to signs of difficulty among my colleagues." "Be more vigilant to signs of distress among colleagues." | 61 |
| Approach, support, or help the colleague | "I will help my peer." "Non-medical human approach." "I think I will have a more compassionate approach towards colleagues in difficulty." | 47 |
| More available or listen more | "Be more listening." "Be more present for them." "Be even more listening towards my colleagues and do not hesitate to ask about their well-being." | 23 |
| Be proactive or take action | "We must not wait." "Be more proactive in approaching a colleague in difficulty." "It is essential to act quickly." | 16 |
| Do not hesitate to act or have the courage to act | "I will not hesitate to help a colleague in difficulty." "One must have the courage to approach a colleague in difficulty." "Do not hesitate to approach a colleague in difficulty." | 8 |
| Ask questions to colleagues, discuss | "I will try to question a colleague who seems to be having difficulties more quickly." "I think I will make the effort more often to have real conversations with my colleagues." "I will be more alert to signs of fatigue among my colleagues and will inquire more about their situation." | 6 |
| Take the time | "Go beyond the taboo and take the time to approach a colleague in difficulty." "Be alert to signs of distress and take the time to be human for a compassionate approach." "Be more open to signs of distress and take the time." | 5 |
| Set boundaries in the helping relationship | "Help my colleagues and respect myself." "Allow myself to approach a colleague in difficulty but avoid getting too involved in trying to find solutions." "Be careful not to take on others' problems as my own... but anything is better than doing nothing." | 3 |
| Use the tools provided by the training | "Use the proposed tools." "Use communication tools." "Approach a colleague in difficulty (with the right tools!)." | 3 |

^(a)^ In total, 153 participants provided verbatims describing their behavioral intention. Categories are non-exclusive.

*Table S2.* Reasons for intention not to change practice

| Reasons for intention not to change practice | Representative excerpts of quotations. | n=28 ^(a)^ |
| --- | --- | --- |
| Past behavior or habit related to behavior | "It was already in my concerns to help a colleague if necessary."  "I’m already doing it."  "Confirms my practice." | 14 |
| Environmental context and resources | "…however, I’m now in solo practice."  "I don't see any at the moment."  "I am changing environments." | 4 |
| Semantic disagreement | "Does not apply to the practice, but rather to the work environment." "Not related to the practice."  "No change to my medical practice. However, I will try to pay more attention to the state of my colleagues." | 3 |
| Beliefs about capabilities | "I do not feel capable of supporting my colleagues at the moment, being a bit overwhelmed by events myself." | 1 |
| Moral norm | "Because it is an intrusion into someone else's life. I would do it for someone close to me but not for all colleagues. It also depends on the type of distress. Some subjects are very difficult to approach." | 1 |
| Self-care or taking care of oneself first | "Because you have to start by taking care of yourself." | 1 |

^(a)^ In total, 28 participants provided verbatims describing reasons for intention not to change practice.

*Table S3.* Barriers and determinants for not adopting behavior, self-administered questionnaire 4 months after course

| **Barrier Category Sub-category** | | **Representative exerpts of quotations** | n=33 ^(a)^ | **%** |
| --- | --- | --- | --- | --- |
| **Environmental context and resources** | No colleagues experiencing difficulties or showing signs of difficulties. | "I did not meet any colleagues in difficulty." | 12 | 36.4% |
|  | No opportunity to adopt behavior. | "Didn't have the opportunity."  "The opportunity didn't arise."  "Didn't have the chance." | 11 | 33.3% |
|  | Not being actively in practice or in full-time practice | "I am on maternity leave."  "I am no longer practicing."  "I work one day a month and only at the office." | 6 | 18.2% |
| **Other** | No need to adopt behavior. | "It wasn't necessary."  "Didn't need to."  "I didn't feel the need for it." | 3 | 9.1% |
|  | Conflict between coworkers | "I didn't want to seem like I was taking sides." | 1 | 3.0% |

^(a)^ In total, 33 participants provided verbatims describing determinants for not adopting behavior 4 months after course.

*Supplementary Graph 1.* Behavioral Domain Targeted by Seven Behavior Change Techniques Present in the CPD Course
